# Metabolites of Siamenoside I and Their Distributions in Rats

**DOI:** 10.3390/molecules21020176

**Published:** 2016-01-30

**Authors:** Xue-Rong Yang, Feng Xu, Dian-Peng Li, Feng-Lai Lu, Guang-Xue Liu, Lei Wang, Ming-Ying Shang, Yong-Lin Huang, Shao-Qing Cai

**Affiliations:** 1 Guangxi Key Laboratory of Functional Phytochemicals Research and Utilization, Guangxi Institute of Botany, Guangxi Zhuang Autonomous Region and Chinese Academy of Sciences, No. 85, Yanshan Road, Yanshan District, Guilin 541006, China; yxrxl@sina.cn (X.-R.Y.); lufenglai@126.com (F.-L.L.); ww8304@126.com (L.W.); hyl@gxib.cn (Y.-L.H.); 2 State Key Laboratory of Natural and Biomimetic Drugs, School of Pharmaceutical Sciences, Peking University, No. 38 Xueyuan Road, Beijing 100191, China; guangxl@bjmu.edu.cn (G.-X.L.); myshang@bjmu.edu.cn (M.-Y.S.); sqcai@bjmu.edu.cn (S.-Q.C.)

**Keywords:** *Siraitia grosvenorii*, mogrosides, siamenoside I, metabolism, distribution, LC-IT-TOF-MS^n^, natural sweeteners, saponins, cucurbitanes

## Abstract

Siamenoside I is the sweetest mogroside that has several kinds of bioactivities, and it is also a constituent of Siraitiae Fructus, a fruit and herb in China. Hitherto the metabolism of siamenoside I in human or animals remains unclear. To reveal its metabolic pathways, a high-performance liquid chromatography-electrospray ionization-ion trap-time of flight-multistage mass spectrometry (HPLC-ESI-IT-TOF-MS^n^) method was used to profile and identify its metabolites in rats. Altogether, 86 new metabolites were identified or tentatively identified, and 23 of them were also new metabolites of mogrosides. In rats, siamenoside I was found to undergo deglycosylation, hydroxylation, dehydrogenation, deoxygenation, isomerization, and glycosylation reactions. Among them, deoxygenation, pentahydroxylation, and didehydrogenation were novel metabolic reactions of mogrosides. The distributions of siamenoside I and its 86 metabolites in rat organs were firstly reported, and they were mainly distributed to intestine, stomach, kidney, and brain. The most widely distributed metabolite was mogroside IIIE. In addition, eight metabolites were bioactive according to literature. These findings would help to understand the metabolism and effective forms of siamenoside I and other mogrosides *in vivo*.

## 1. Introduction

Mogrosides are a group of cucurbitane-type triterpenoid saponins which have the common aglycone of mogrol [1]. They are responsible for the sweet taste and bioactivities of Siraitiae Fructus (Luo Han Guo in Chinese, the ripe fruits of *Siraitia grosvenorii*), a traditional Chinese medicine and an edible fruit [2].

Siamenoside I is one of the mogrosides, which is firstly isolated from *Siraitia siamensis* (a Chinese folk medicine) [3] and then from *Siraitia grosvenorii* [4]. Its relative sweetness (0.01% solution) to 5% sucrose is determined to be 563, higher than the famous sweetener mogroside V, making it the sweetest cucurbitane glycoside [4].

Besides its intense sweet taste, siamenoside I also has several kinds of bioactivities. It can inhibit the induction of Epstein–Barr virus early antigen (EBV-EA) by 12-*O*-tetradecanoylphorbol-13-acetate (TPA) in Raji cells, which implies that it is a potential cancer chemopreventive agent [5]. It also inhibits two-stage carcinogenesis induced by 9,10-dimethyl-1,2-benzanthracene (DMBA) and TPA in mice [6]. Furthermore, it exhibits a maltase inhibitory effect with IC_50_ value of 10 mM, which is more potent than those of mogroside V and mogroside IV (IC_50_ of 14 mM and 12 mM, respectively) [7]. 

In order to clarify the action mechanisms of the beneficial effects of mogrosides and to develop them into new health foods or drugs or sweeteners, it is necessary to investigate their metabolism and disposition. Up to now, there are only three reports on the metabolism of mogrosides. The first is about the human intestinal microflora biotransformation of mogroside III [8]; the second is on the *in vivo* digestion, absorption and metabolism of 72% mogroside V in rats [9], and the third is our study on the *in vitro*, *in vivo* metabolism of mogroside V (purity >98%) and the distributions of its metabolites in rats [10]. We find that mogroside V can be metabolized to its secondary glycosides and the aglycone morgol, and then morgol is oxidized to lots of metabolites. However, there are no reports on the metabolism of siamenoside I so far.

Although the importance of studying drug distribution in various organs is well established in the drug development field, the studies on the distributions of metabolites of bioactive natural products are neglected. Since our previous research indicates that the metabolites of natural products distribute unevenly in different organs of rats, such as mogroside V [10] and (+)-catechin [11], we believe that revealing the distributions of a bioactive natural product and its metabolites can be helpful in understanding its target organ and organ-specific bioactivities.

Accordingly, in the present work, the metabolites of siamenoside I and the distributions of siamenoside I and its metabolites in rats were firstly investigated by high-performance liquid chromatography-electrospray ionization-ion trap-time of flight-multistage mass spectrometry (HPLC-ESI-IT-TOF-MS^n^). In total, 86 new metabolites of siamenoside I in rats were detected and identified or tentatively identified, and the metabolic pathways and *in vivo* processes of siamenoside I were proposed. Siamenoside I and its metabolites were mainly distributed to intestine, stomach, kidney, and brain, and mogroside IIIE was the most widely distributed metabolite.

## 2. Results

### 2.1. Profiling the Metabolites of Siamenoside I in Different Biosamples by HPLC-ESI-IT-TOF-MS^n^

Based on the strategy described in Section 4.7, 86 new metabolites (**M1**–**M86**) of siamenoside I were detected altogether in different drug-containing samples by the HPLC-ESI-IT-TOF-MS^n^ technique (Table 1, Table S1, Figure 1, and Figures S1–S25).

Eighty-three metabolites were detected in drug-containing feces; 19 metabolites were found in urine, and only two were detected in plasma.

As for different organs, 2, 7, 7, 3, 13, 21, 19, and 14 metabolites were detected in heart, liver, spleen, lungs, kidneys, stomach, intestine, and brain, respectively. Furthermore, no metabolites were detected in muscles.

### 2.2. Identification of the Metabolites of Siamenoside I in Different Biosamples by HPLC-ESI-IT-TOF-MS^n^

Nine new metabolites of siamenoside I were unambiguously identified to be mogroside IVA (**M3**), mogroside IVE (**M4**), mogroside III (**M8**), mogroside IIIE (**M9**), mogroside IIIA_1_ (**M10**), mogroside IIE (**M15**), mogroside IIA_2_ (**M17**), 11-oxomogroside IIE (**M20**), and mogrol (**M29**) sequentially by comparison of their LC-MS^n^ data to those of reference compounds.

The other 77 metabolites were tentatively identified by interpretation of their LC-MS^n^ data and by comparison with literature.

These 86 new metabolites of siamenoside I can be classified into 24 classes according to their formative reactions and molecular formulae.

**Table 1:** LC-MS data of siamenoside I and its 86 metabolites formed in rats and their formative reactions.

No.	t_R_ (min)	Meas. (Da)	Pred.(Da)	Err. (ppm)	DBE ^2^	Formula	Identification	Reactions
**M0 ^1^**	24.966	1169.5959	1169.5961	0.17	9	C_54_H_92_O_24_	siamenoside I	−
**M1**	25.698	1285.6444	1285.6434	0.78	10	C_60_H_102_O_29_	mogroside V isomer	+Glc
**M2**	26.043	1285.6441	1285.6434	0.54	10	C_60_H_102_O_29_	mogroside V isomer	+Glc
**M3 ^1^**	25.345	1169.5905	1169.5961	−0.09	9	C_54_H_92_O_24_	mogroside IVA	isomerization
**M4 ^1^**	25.960	1169.5923	1169.5961	−3.25	9	C_54_H_92_O_24_	mogroside IVE	isomerization
**M5**	26.696	1169.5950	1169.5961	−0.94	9	C_54_H_92_O_24_	mogroside IV isomer	isomerization
**M6**	25.923	1167.5730	1167.5804	−2.23	10	C_54_H_90_O_24_	dehydrogenated siamenoside I	−2H
**M7**	25.983	1153.5957	1153.6011	−4.25	9	C_54_H_92_O_23_	deoxygenated siamenoside I	−O
**M8 ^1^**	26.803	1007.5425	1007.5432	−0.69	8	C_48_H_82_O_19_	mogroside III	−Glc
**M9 ^1^**	27.173	1007.5387	1007.5432	−4.47	8	C_48_H_82_O_19_	mogroside IIIE	−Glc
**M10 ^1^**	30.217	1007.5376	1007.5432	−5.56	8	C_48_H_82_O_19_	mogroside IIIA_1_	−Glc
**M11**	30.525	1007.5395	1007.5432	−3.67	8	C_48_H_82_O_19_	mogroside III isomer	−Glc
**M12**	30.940	1007.5385	1007.5432	−4.66	8	C_48_H_82_O_19_	mogroside III isomer	−Glc
**M13**	26.927	1005.5269	1005.5276	0.70	9	C_48_H_80_O_19_	dehydrogenated mogroside III isomer	−Glc − 2H
**M14**	27.728	991.5441	991.5483	−4.24	8	C_48_H_82_O_18_	deoxygenated mogroside III isomer	−Glc − O
**M15 ^1^**	29.365	845.4881	845.4904	−2.72	7	C_42_H_72_O_14_	mogroside IIE	−2Glc
**M16**	30.648	845.4944	845.4904	4.73	7	C_42_H_72_O_14_	mogroside II isomer	−2Glc
**M17 ^1^**	31.775	845.4910	845.4904	0.71	7	C_42_H_72_O_14_	mogroside IIA_2_	−2Glc
**M18**	33.298	845.4884	845.4904	−2.37	7	C_42_H_72_O_14_	mogroside II isomer	−2Glc
**M19**	33.905	845.4918	845.4904	1.66	7	C_42_H_72_O_14_	mogroside II isomer	−2Glc
**M20 ^1^**	29.908	843.4737	843.4748	−1.30	8	C_42_H_70_O_14_	11-oxomogroside IIE	−2Glc − 2H
**M21**	33.604	843.4726	843.4748	−2.61	8	C_42_H_70_O_14_	dehydrogenated mogroside II isomer	−2Glc − 2H
**M22**	34.028	829.4946	829.4955	−1.08	7	C_42_H_72_O_13_	deoxygenated mogroside II isomer	−2Glc − O
**M23**	34.813	827.4780	827.4798	−2.18	8	C_42_H_70_O_13_	dehydrogenated deoxygenated mogroside II isomer	−2Glc − 2H − O
**M24**	34.997	683.4348	683.4376	−1.76	6	C_36_H_62_O_9_	mogroside IA_1_	−3Glc
**M25**	37.507	683.4366	683.4376	1.46	6	C_36_H_62_O_9_	mogroside IE_1_	−3Glc
**M26**	36.120	681.4196	681.4219	−3.38	7	C_36_H_60_O_9_	dehydrogenated mogroside I isomer	−3Glc − 2H
**M27**	38.860	681.4215	681.4219	−0.59	7	C_36_H_60_O_9_	dehydrogenated mogroside I isomer	−3Glc − 2H
**M28**	45.978	521.3834	521.3848	−2.69	5	C_30_H_52_O_4_	mogrol isomer	−4Glc
**M29 ^1^**	46.467	521.3838	521.3848	−1.92	5	C_30_H_52_O_4_	mogrol	−4Glc
**M30**	52.478	519.3676	519.3691	−2.89	6	C_30_H_50_O_4_	dehydrogenated mogrol	−4Glc − 2H
**M31**	52.953	519.3687	519.3691	−0.77	6	C_30_H_50_O_4_	dehydrogenated mogrol	−4Glc − 2H
**M32**	23.007	553.3721	553.3746	−4.52	5	C_30_H_52_O_6_	dihydroxylated mogrol	−4Glc + 2O
**M33**	26.528	553.3699	553.3746	−8.49	5	C_30_H_52_O_6_	dihydroxylated mogrol	−4Glc + 2O
**M34**	27.482	553.3717	553.3746	−5.24	5	C_30_H_52_O_6_	dihydroxylated mogrol	−4Glc + 2O
**M35**	28.152	553.3740	553.3746	−1.08	5	C_30_H_52_O_6_	dihydroxylated mogrol	−4Glc + 2O
**M36**	26.475	551.3559	551.3589	−5.40	6	C_30_H_50_O_6_	dehydrogenated dihydroxylated mogrol	−4Glc − 2H + 2O
**M37**	27.050	551.3557	551.3589	−5.80	6	C_30_H_50_O_6_	dehydrogenated dihydroxylated mogrol	−4Glc − 2H + 2O
**M38**	29.487	551.3566	551.3589	−4.17	6	C_30_H_50_O_6_	dehydrogenated dihydroxylated mogrol	−4Glc − 2H + 2O
**M39**	30.887	551.3558	551.3589	−5.62	6	C_30_H_50_O_6_	dehydrogenated dihydroxylated mogrol	−4Glc − 2H + 2O
**M40**	31.537	551.3561	551.3589	−5.04	6	C_30_H_50_O_6_	dehydrogenated dihydroxylated mogrol	−4Glc − 2H + 2O
**M41**	33.122	551.3549	551.3589	−7.25	6	C_30_H_50_O_6_	dehydrogenated dihydroxylated mogrol	−4Glc − 2H + 2O
**M42**	16.305	569.3655	569.3695	−7.03	5	C_30_H_52_O_7_	trihydroxylated mogrol	−4Glc + 3O
**M43**	16.728	569.3671	569.3695	−4.22	5	C_30_H_52_O_7_	trihydroxylated mogrol	−4Glc + 3O
**M44**	17.280	569.3660	569.3695	−6.15	5	C_30_H_52_O_7_	trihydroxylated mogrol	−4Glc + 3O
**M45**	18.140	569.3674	569.3695	−3.69	5	C_30_H_52_O_7_	trihydroxylated mogrol	−4Glc + 3O
**M46**	18.923	569.3675	569.3695	−3.51	5	C_30_H_52_O_7_	trihydroxylated mogrol	−4Glc + 3O
**M47**	21.464	569.3675	569.3695	−3.51	5	C_30_H_52_O_7_	trihydroxylated mogrol	−4Glc + 3O
**M48**	21.755	569.3674	569.3695	−3.69	5	C_30_H_52_O_7_	trihydroxylated mogrol	−4Glc + 3O
**M49**	22.121	569.3666	569.3695	−5.09	5	C_30_H_52_O_7_	trihydroxylated mogrol	−4Glc + 3O
**M50**	22.531	569.3667	569.3695	−7.03	5	C_30_H_52_O_7_	trihydroxylated mogrol	−4Glc + 3O
**M51**	11.410	567.3489	567.3539	−8.80	6	C_30_H_50_O_7_	dehydrogenated trihydroxylated mogrol	−4Glc − 2H + 3O
**M52**	18.482	567.3463	567.3539	−13.4	6	C_30_H_50_O_7_	dehydrogenated trihydroxylated mogrol	−4Glc − 2H + 3O
**M53**	19.893	567.3534	567.3539	−2.64	6	C_30_H_50_O_7_	dehydrogenated trihydroxylated mogrol	−4Glc − 2H + 3O
**M54**	20.977	567.3494	567.3539	−0.88	6	C_30_H_50_O_7_	dehydrogenated trihydroxylated mogrol	−4Glc − 2H + 3O
**M55**	21.761	567.3512	567.3539	−7.93	6	C_30_H_50_O_7_	dehydrogenated trihydroxylated mogrol	−4Glc − 2H + 3O
**M56**	22.365	567.3507	567.3539	−4.76	6	C_30_H_50_O_7_	dehydrogenated trihydroxylated mogrol	−4Glc − 2H + 3O
**M57**	24.123	567.3497	567.3539	−5.64	6	C_30_H_50_O_7_	dehydrogenated trihydroxylated mogrol	−4Glc − 2H + 3O
**M58**	24.478	567.3508	567.3539	−7.23	6	C_30_H_50_O_7_	dehydrogenated trihydroxylated mogrol	−4Glc − 2H + 3O
**M59**	25.380	567.3499	567.3539	−5.46	6	C_30_H_50_O_7_	dehydrogenated trihydroxylated mogrol	−4Glc − 2H + 3O
**M60**	27.050	567.3498	567.3539	−7.05	6	C_30_H_50_O_7_	dehydrogenated trihydroxylated mogrol	−4Glc − 2H + 3O
**M61**	27.728	567.3520	567.3539	−7.23	6	C_30_H_50_O_7_	dehydrogenated trihydroxylated mogrol	−4Glc − 2H + 3O
**M62**	24.412	565.3351	565.3382	−5.48	7	C_30_H_48_O_7_	didehydrogenated trihydroxylated mogrol	−4Glc − 4H + 3O
**M63**	26.052	565.3357	565.3382	−4.42	7	C_30_H_48_O_7_	didehydrogenated trihydroxylated mogrol	−4Glc − 4H + 3O
**M64**	28.810	565.3368	565.3382	−2.48	7	C_30_H_48_O_7_	didehydrogenated trihydroxylated mogrol	−4Glc − 4H + 3O
**M65**	30.340	565.3347	565.3382	−6.19	7	C_30_H_48_O_7_	didehydrogenated trihydroxylated mogrol	−4Glc − 4H + 3O
**M66**	13.872	585.3613	585.3644	−5.30	5	C_30_H_52_O_8_	tetrahydroxylated mogrol	−4Glc + 4O
**M67**	14.242	585.3601	585.3644	−7.35	5	C_30_H_52_O_8_	tetrahydroxylated mogrol	−4Glc + 4O
**M68**	14.603	585.3608	585.3644	−6.15	5	C_30_H_52_O_8_	tetrahydroxylated mogrol	−4Glc + 4O
**M69**	18.307	585.3603	585.3644	−4.95	5	C_30_H_52_O_8_	tetrahydroxylated mogrol	−4Glc + 4O
**M70**	19.408	585.3613	585.3644	−5.30	5	C_30_H_52_O_8_	tetrahydroxylated mogrol	−4Glc + 4O
**M71**	15.573	583.3444	583.3488	−7.54	6	C_30_H_50_O_8_	dehydrogenated tetrahydroxylated mogrol	−4Glc − 2H + 4O
**M72**	15.997	583.3454	583.3488	−8.54	6	C_30_H_50_O_8_	dehydrogenated tetrahydroxylated mogrol	−4Glc − 2H + 4O
**M73**	20.492	583.3454	583.3488	−6.00	6	C_30_H_50_O_8_	dehydrogenated tetrahydroxylated mogrol	−4Glc − 2H + 4O
**M74**	20.800	583.3465	583.3488	−3.94	6	C_30_H_50_O_8_	dehydrogenated tetrahydroxylated mogrol	−4Glc − 2H + 4O
**M75**	21.453	583.3465	583.3488	−3.94	6	C_30_H_50_O_8_	dehydrogenated tetrahydroxylated mogrol	−4Glc − 2H + 4O
**M76**	22.895	583.3452	583.3488	−6.17	6	C_30_H_50_O_8_	dehydrogenated tetrahydroxylated mogrol	−4Glc − 2H + 4O
**M77**	24.710	583.3447	583.3488	−7.03	6	C_30_H_50_O_8_	dehydrogenated tetrahydroxylated mogrol	−4Glc − 2H + 4O
**M78**	20.615	581.3297	581.3331	−5.85	7	C_30_H_48_O_8_	didehydrogenated tetrahydroxylated mogrol	−4Glc − 4H + 4O
**M79**	21.815	581.3290	581.3331	−7.05	7	C_30_H_48_O_8_	didehydrogenated tetrahydroxylated mogrol	−4Glc − 4H + 4O
**M80**	23.007	581.3292	581.3331	−6.71	7	C_30_H_48_O_8_	didehydrogenated tetrahydroxylated mogrol	−4Glc − 4H + 4O
**M81**	23.433	581.3306	581.3331	−4.03	7	C_30_H_48_O_8_	didehydrogenated tetrahydroxylated mogrol	−4Glc − 4H + 4O
**M82**	23.988	581.3308	581.3331	−3.96	7	C_30_H_48_O_8_	didehydrogenated tetrahydroxylated mogrol	−4Glc − 4H + 4O
**M83**	25.682	581.3304	581.3331	−4.64	7	C_30_H_48_O_8_	didehydrogenated tetrahydroxylated mogrol	−4Glc − 4H + 4O
**M84**	25.990	581.3291	581.3331	−4.00	7	C_30_H_48_O_8_	didehydrogenated tetrahydroxylated mogrol	−4Glc − 4H + 4O
**M85**	17.707	587.2977	587.2992	−2.55	7	C_30_H_48_O_9_	didehydrogenated pentahydroxylated mogrol	−4Glc − 4H + 5O
**M86**	18.607	587.2983	587.2992	−1.53	7	C_30_H_48_O_9_	didehydrogenated pentahydroxylated mogrol	−4Glc − 4H + 5O

^1^ Confirmed by comparison with reference compounds. ^2^ DBE, double bond equivalent.

#### 2.2.1. Metabolites Formed by Monoglycosylation (**M1**, **M2**)

**M1**–**M2** showed [M + HCOOH − H]^−^ at *m*/*z* 1285.64, which indicated that their molecular formulae were C_60_H_102_O_29_. The formulae had an additional glucosyl (C_6_H_10_O_5_) than that of siamenoside I (C_54_H_92_O_24_). Hence, they were mogroside V isomers.

**Figure 1:**
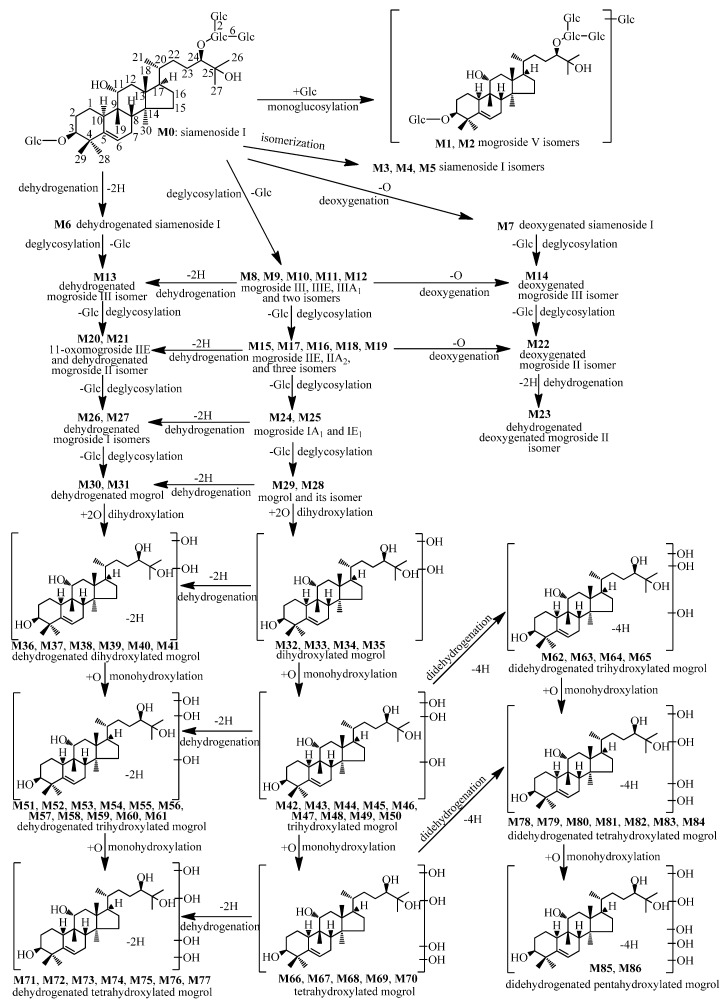
The proposed metabolic pathways of siamenoside I in rats.

#### 2.2.2. Metabolites Formed by Isomerization (**M3**–**M5**)

**M3**–**M5** showed [M + HCOOH − H]^−^ at *m*/*z* 1169.59, indicating the molecular formula of C_54_H_92_O_24_, which was the same to siamenoside I. Hence, they were mogroside IV isomers, and **M3**–**M4** were further confirmed as mogroside IVA and mogroside IVE by comparison with reference compounds.

#### 2.2.3. Metabolites Formed by Dehydrogenation (**M6**)

The molecular formula of **M6** was predicted to be C_54_H_9__0_O_24_ based on its [M + HCOOH − H]^−^ at *m*/*z* 1167.5730, which was formed by loss of two hydrogen atoms from siamenoside I, thus **M6** was tentatively identified as dehydrogenated siamenoside I.

#### 2.2.4. Metabolites Formed by Deoxygenation (**M7**)

The molecular formula of **M7** was C_54_H_92_O_2__3_ calculated from its [M + HCOOH − H]^−^ at *m*/*z* 1153.5957, which has one less oxygen atom than that of siamenoside I. Accordingly, it was tentatively identified as deoxygenated siamenoside I.

#### 2.2.5. Metabolites Formed by Deglucosylation (**M8**–**M12**)

**M8**–**M12** showed [M + HCOOH − H]^−^ at *m*/*z* 1007.54, implying their molecular formulae of C_48_H_82_O_19_. The formulae had one less glucosyl group (element composition: C_6_H_10_O_5_) than that of siamenoside I, so they were mogroside III isomers. In addition, **M8**–**M10** were unambiguously identified as mogroside III, mogroside IIIE, and mogroside IIIA_1_ by comparison with reference compounds.

#### 2.2.6. Metabolites Formed by Deglucosylation and Dehydrogenation (**M13**)

**M13** had the molecular formula of C_48_H_80_O_19_ predicted by its [M + HCOOH − H]^−^ at *m*/*z* 1005.5269. Compared with C_48_H_82_O_19_ of mogroside III isomers, it was tentatively identified as dehydrogenated mogroside III isomer. Moreover, in the MS^2^ of **M13**, [M − H − 2Glc]^−^ at *m*/*z* 797.4629 (C_42_H_69_O_14_), [M −H − 3Glc]^−^ at *m*/*z* 635.4090 (C_36_H_59_O_9_), [aglycon − H]^−^ at *m*/*z* 473.3623 (C_30_H_49_O_4_) were observed. Hence, **M13** was a triglucoside of dehydrogenated mogrol.

#### 2.2.7. Metabolites Formed by Deglucosylation and Deoxygenation (**M14**)

The molecular formula of **M14** was calculated to be C_48_H_8__2_O_1__8_ by its [M + HCOOH − H]^−^ at *m*/*z* 991.5441, which had one less oxygen atom than C_48_H_82_O_19_ of mogroside III, so it was tentatively identified as deoxygenated mogroside III isomer.

#### 2.2.8. Metabolites Formed By Dideglucosylation (**M15**–**M19**)

The molecular formulae of **M15**–**M19** were determined to be C_42_H_72_O_14_ based on their [M + HCOOH − H]^−^ at *m*/*z* 845.49, which had one less glucosyl group (element composition: C_6_H_10_O_5_) than C_48_H_82_O_19_ of mogroside III, so they were mogroside II isomers. Furthermore, **M15** and **M17** were confirmed to be mogroside IIE and mogroside IIA_2_ by comparison with reference compounds.

#### 2.2.9. Metabolites Formed by Dideglucosylation and Dehydrogenation (**M20**–**M21**)

**M20**–**M21** showed [M + HCOOH − H]^−^ at *m*/*z* 843.47 in MS, suggesting their molecular formulae of C_42_H_70_O_14_. Additionally, they showed [aglycon − H]^−^ at *m*/*z* 473.3623 (C_30_H_49_O_4_) in MS^2^ spectra. Therefore, they were tentatively identified as dehydrogenated mogroside II isomer, *i.e.*, diglucoside of dehydrogenated mogrol. Further, **M20** was unambiguously identified as 11-oxomogroside IIE by comparison with reference compounds.

#### 2.2.10. Metabolites Formed by Dideglucosylation and Deoxygenation (**M22**)

**M22** was tentatively identified as deoxygenated mogroside II isomer, since its molecular formula was determined to be C_42_H_72_O_13_ by its [M + HCOOH − H]^−^ at *m*/*z* 829.4946, which had one less oxygen atom than C_42_H_72_O_14_ of mogroside II isomers. In addition, **M22** showed [M − H]^−^ at *m*/*z* 783.4833 (C_42_H_71_O_13_), [M−H−C_6_H_10_O_4_ (deoxyhexosyl)]^−^ at *m*/*z* 637.4252 (C_36_H_61_O_9_), and [M−H−C_6_H_10_O_4_−Glc]^−^ at *m*/*z* 475.3742 (C_30_H_51_O_4_) in MS^2^ spectra, which indicated that the deoxygenation occurred in hexose and the aglycone was mogrol. Thus, **M22** was identified as a deoxyhexosyl-glucosyl mogrol.

#### 2.2.11. Metabolites Formed by Dideglucosylation, Dehydrogenation, and Deoxygenation (**M23**)

The molecular formula of **M23** was determined to be C_42_H_70_O_13_ according to its [M + HCOOH − H]^−^ at *m*/*z* 827.4780, which lost two hydrogen atoms from C_42_H_72_O_13_ of **M22**. Consequently, **M23** was tentatively identified as a dehydrogenated deoxygenated mogroside II isomer.

#### 2.2.12. Metabolites Formed by Trideglucosylation (**M24**–**M25**)

**M24**–**M25** showed [M + HCOOH − H]^−^ at *m*/*z* 683.43 in MS, and [M−H]^−^ at *m*/*z* 637.42 (C_36_H_61_O_9_), [aglycone−H]^−^ at *m*/*z* 475.37 (C_30_H_51_O_4_) in MS^2^ spectra, which implied that they were mogrol glucoside. By comparison with the LC-MS^n^ data in literature [10], **M24** and **M25** were tentatively identified as mogroside IA_1_ and mogroside IE_1_, respectively.

#### 2.2.13. Metabolites Formed by Trideglucosylation and Dehydrogenation (**M26**–**M27**)

The molecular formulae of **M26**–**M27** were predicted to be C_36_H_6__0_O_9_ based on its [M + HCOOH − H]^−^ at *m*/*z* 681.42 in MS, which had two less hydrogen atoms than C_36_H_62_O_9_ of mogroside I isomers. In their negative ion (NI) MS^2^ spectra, [M − H]^−^ at *m*/*z* 635.41 (C_36_H_59_O_9_) and [aglycone−H]^−^ at *m*/*z* 473.36 (C_30_H_49_O_4_) were detected. As a result, **M26**–**M27** were tentatively identified as glucosides of dehydrogenated mogrol, *i.e.*, dehydrogenated mogroside I isomers.

#### 2.2.14. Metabolites Formed by Tetradeglucosylation (**M28**–**M29**)

**M29** was unambiguously identified as mogrol by comparison with reference compound. **M28** had the same molecular formula to mogrol, which showed [M + HCOOH − H]^−^ at *m*/*z* 521.38 in MS, so it was a mogrol isomer.

#### 2.2.15. Metabolites Formed by Tetradeglucosylation and Dehydrogenation (**M30**–**M31**)

**M30** and **M31** had the molecular formulae of C_30_H_50_O_4_ predicted by their [M + HCOOH − H]^−^ at *m*/*z* 519.36, which had two less hydrogen atoms than C_30_H_52_O_4_ of mogrol, thus they were tentatively identified as dehydrogenated mogrols.

#### 2.2.16. Metabolites Formed by Tetradeglucosylation and Dihydroxylation (**M32**–**M35**)

**M32**–**M35** showed [M + HCOOH − H]^−^ at *m*/*z* 553.37, indicating the molecular formula of C_30_H_52_O_6_. Compared to C_30_H_52_O_4_ of mogrol, it had two more oxygen atoms. Accordingly, **M32**–**M35** were tentatively identified as dihydroxylated mogrols.

**Figure 2:**
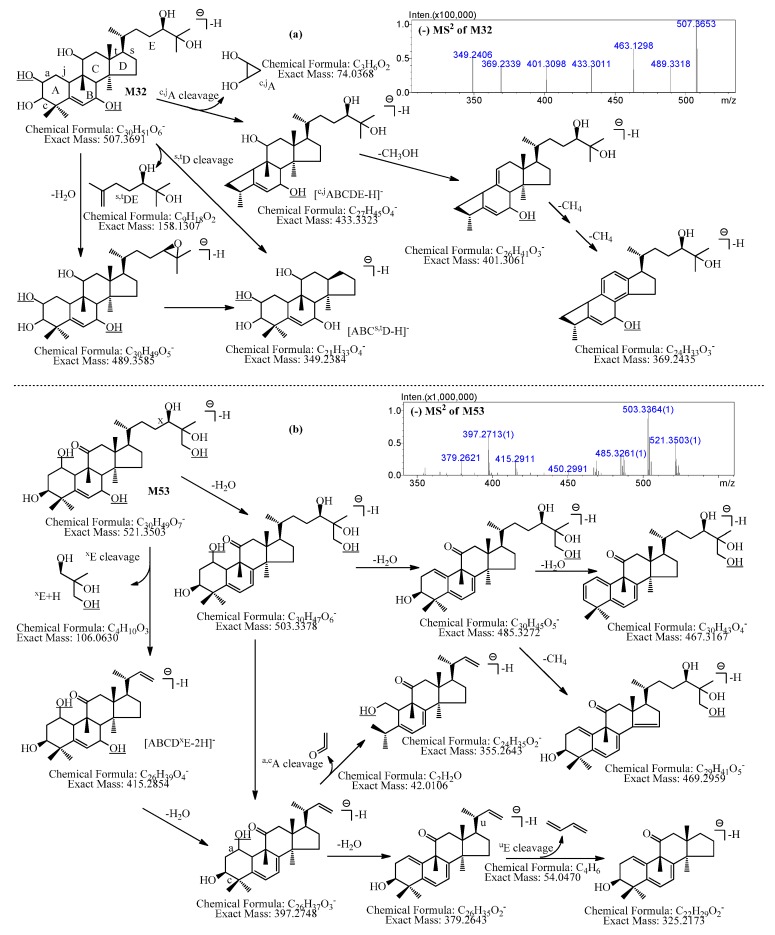
The MS^2^ spectra, characteristic fragment ions, and proposed fragmentation pathways of **M32** and **M53**. (**a**) **M32**; (**b**) **M53**.

In addition, the possible hydroxylation sites of **M32** can be deduced by its MS^2^ data and one possible structure of **M32** is shown in Figure 2a. The nomenclature for the fragmentation pathways and fragment ions of cucurbitanes proposed by the authors [10] were used in this study.

In MS^2^ spectra of **M32**, *m*/*z* 433.3011 ([^c,j^ABCDE − H]^−^, C_27_H_45_O_4_) generated by ^c,j^A cleavage and *m*/*z* 349.2406 ([^s,t^DE − H]^−^, C_21_H_33_O_4_) generated by ^s,t^D cleavage were observed, which indicated that one hydroxylation site was in ^c,j^A, and the other was in ^c,j^ABC^s,t^D (Figure 2a).

#### 2.2.17. Metabolites Formed by Tetradeglucosylation, Dihydroxylation, and Dehydrogenation (**M36**–**M41**)

The molecular formulae of **M36**–**M41** were calculated to be C_30_H_50_O_6_ based on their [M + HCOOH − H]^−^ at *m*/*z* 551.35. Compared to C_30_H_52_O_6_ of **M32**–**M35**, it had two less hydrogen atoms. Accordingly, **M36**–**M41** were tentatively identified as dehydrogenated dihydroxylated mogrols.

#### 2.2.18. Metabolites Formed by Tetradeglucosylation and Trihydroxylation (**M42**–**M50**)

**M42**–**M50** had the molecular formulae of C_30_H_52_O_7_ predicted by their [M + HCOOH − H]^−^ at *m*/*z* 569.36. Compared to C_30_H_52_O_6_ of **M32**–**M35** (dihydroxylated mogrol), it had one more oxygen atom. Accordingly, **M42**–**M50** were tentatively identified as trihydroxylated mogrols.

#### 2.2.19. Metabolites Formed by Tetradeglucosylation, Trihydroxylation, and Dehydrogenation (**M51**–**M61**)

The molecular formulae of **M51**–**M61** were determined to be C_30_H_50_O_7_ on the basis of their [M + HCOOH − H]^−^ at *m*/*z* 567.35. In comparison with C_30_H_52_O_7_ of **M42**–**M50** (trihydroxylated mogrol), it had two less hydrogen atoms. As a result, **M51**–**M61** were tentatively identified as dehydrogenated trihydroxylated mogrols.

Further, the possible hydroxylation sites of **M53** could be deduced by its MS^2^ data, and one possible structure of **M53** is shown in Figure 2b.

**M53** showed [M + HCOOH − H]^−^ at *m*/*z* 567.3524 in MS, and then it was fragmented into [M − H]^−^ at *m*/*z* 521.3207 (C_30_H_49_O_7_) in MS^2^ spectrum. The [M − H]^−^ was further cleaved into product ions at *m*/*z* 503.3364 (C_30_H_47_O_6_), 485.3261 (C_30_H_45_O_5_), and 467.3056 (C_30_H_43_O_4_) formed by sequential losses of H_2_O. It was also cleaved into product ion at *m*/*z* 415.2911 (C_26_H_39_O_4_) by losing C_4_H_10_O_3_ (^x^E+H), which indicated that one hydroxylation site was in ^x^E. Besides, the characteristic fragment ions at *m*/*z* 397.2713 (C_26_H_37_O_3_), *m*/*z* 379.2621 (C_26_H_35_O_2_), *m*/*z* 355.2613 (C_24_H_35_O_2_), and *m*/*z* 325.2449 (C_22_H_29_O_2_) were observed in MS^2^, indicating that the other two hydroxylation sites should be located at ^a,c^ABCD^u^E (Figure 2b).

#### 2.2.20. Metabolites Formed by Tetradeglucosylation, Trihydroxylation, and Didehydrogenation (**M62**–**M65**)

**M62**-**M65** had the molecular formulae of C_30_H_48_O_7_ predicted by their [M + HCOOH − H]^−^ at *m*/*z* 565.33, which had two less hydrogen atoms than C_30_H_50_O_7_ of **M51**–**M61**. Accordingly, **M62**–**M65** were tentatively identified as didehydrogenated trihydroxylated mogrols.

#### 2.2.21. Metabolites Formed by Tetradeglucosylation and Tetrahydroxylation (**M66**–**M70**)

**M66**–**M70** showed [M + HCOOH − H]^−^ at *m*/*z* 585.36, indicating the molecular formula of C_30_H_52_O_8_. Compared with C_30_H_52_O_4_ of mogrol, their molecular formula had four more oxygen atoms. Therefore, they were tetrahydroxylated mogrols.

#### 2.2.22. Metabolites Formed by Tetradeglucosylation, Tetrahydroxylation, and Dehydrogenation (**M71**–**M77**)

The molecular formulae of **M71**–**M77** were determined to be C_30_H_5__0_O_8_ based on their [M + HCOOH − H]^−^ at *m*/*z* 583.34, which had two less hydrogen atoms than C_30_H_52_O_8_ of **M66**–**M70** (tetrahydroxylated mogrol). Accordingly, they were tentatively identified as dehydrogenated tetrahydroxylated mogrol.

**Figure 3:**
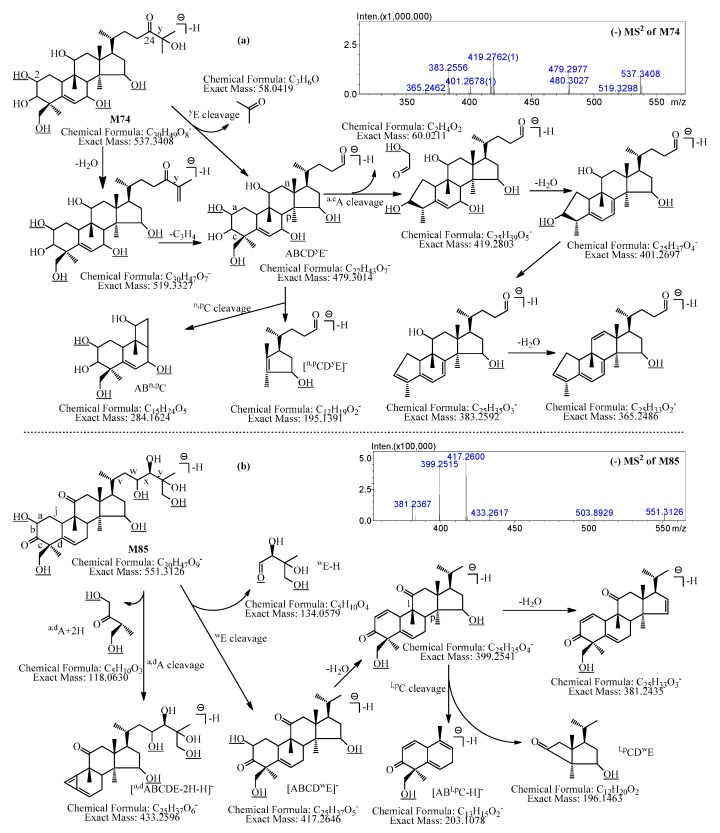
The MS^2^ spectra, characteristic fragment ions, and proposed fragmentation pathways of **M74** and **M85**. (**a**) **M74**; (**b**) **M85**.

Besides, the possible dehydrogenation and hydroxylation sites of **M74** could be deduced by its MS^2^ and MS^3^ data, and one possible structure of **M74** is shown in Figure 3a.

The characteristic product ions at *m*/*z* 479.2977 (C_27_H_43_O_7_, ABCD^y^E^−^) produced by ^y^E cleavage, *m*/*z* 419.2762 (C_25_H_39_O_5_) generated by ^a,c^A cleavage, and *m*/*z* 195.1347 (C_12_H_19_O_2_) generated by ^n,p^C cleavage indicated that the C_24_-hydroxyl group of **M74** was dehydrogenated, and one of the four tetrahydroxylation sites was at C_2_, one was in ^n,p^CD^y^E, and the other two were in AB^n,p^C (Figure 3a).

#### 2.2.23. Metabolites Formed by Tetradeglucosylation, Tetrahydroxylation, and Didehydrogenation (**M78**–**M84**)

**M78**–**M84** showed [M + HCOOH − H]^−^ at *m*/*z* 581.33 in their MS, indicating their molecular formulae of C_30_H_48_O_8_, which had two less hydrogen atoms than C_30_H_50_O_8_ of **M71**–**M77**. Therefore, **M78**–**M84** were tentatively identified to be didehydrogenated tetrahydroxylated mogrol.

#### 2.2.24. Metabolites Formed by Tetradeglucosylation, Pentahydroxylation, and Didehydrogenation (**M85**–**M86**)

**M85**–**M86** had the molecular formulae of C_30_H_48_O_9_ predicted by their [M + HCOOH − H]^−^ at *m*/*z* 587.29 in their MS, which had one more oxygen atom than C_30_H_48_O_8_ of **M78**–**M84**. Accordingly, **M85**–**M86** were tentatively identified as didehydrogenated pentahydroxylated mogrol.

In addition, the possible dehydrogenation and hydroxylation sites of **M85** could be deduced by its MS^2^ and MS^3^ data, and one possible structure of **M85** is shown in Figure 3b.

**M85** showed [M + Cl]^−^ at *m*/*z* 587.2977, which was fragmented into [M − H]^−^ at *m*/*z* 551.3126 (C_30_H_47_O_9_) in MS^2^ spectrum. The [M − H]^−^ was then fragmented into characteristic product ions at *m*/*z* 433.2617 (C_25_H_37_O_6_, [^a,d^ABCDE − 2H − H]^−^) and *m*/*z* 417.2600 (C_25_H_37_O_5_, [ABCD^w^E]^−^) by ^a,d^A cleavage and ^w^E cleavage respectively, which implied that two of the five hydroxylation sites were in ^a,d^A, other two were in ^w^E. Furthermore, the ion at *m*/*z* 417.2600 (C_25_H_37_O_5_, [ABCD^w^E]^−^) was cleaved into product ions at *m*/*z* 399.2515 (C_25_H_35_O_4_) and *m*/*z* 203.0987 (C_13_H_15_O_2_) by sequential loss of H_2_O and ^l,p^CD^w^E (C_12_H_20_O_2_) in MS^3^ spectra, which indicated that the last of the five hydroxylation sites was in ^l,p^CD^w^E.

### 2.3. Distribution of the Metabolites of Siamenoside I in Rats

The peak areas and distributions of siamenoside I and the 86 identified metabolites in different biological samples are shown in Table 2.

**Table 2:** Distribution of siamenoside I and its 86 metabolites in rat organs and their peak areas calculated from extracted ion chromatograms (EICs).

No.	Feces	Urine	Plasma	Heart ^1^	Liver ^1^	Spleen ^1^	Lung ^1^	Kidney ^1^	Stomach ^1^	Intestine ^1^	Brain ^1^	Muscle	TPA ^2^
**M0**	9,489,561	45,194,235	929,711				655,725	1,151,491	15,473,808	1,201,5247	1,162,190		86,071,968
**M1**	1,144,657	9,555,900							2,001,769	1,487,067	680,786		14,870,179
**M2**		15,997,951									531,345		16,529,296
**M3**	760,580	847,869						1,307,673	1,655,097	2,264,310	177,956		7,013,485
**M4**	545,593	5,114,666			2,993,972	1,200,234		2,991,375	1,454,847	2,022,516	66,665		16,389,868
**M5**	1,984,320	2,244,041								3,096,436	301,433		7,626,230
**M6**	1,370,701	24,868,935	522,375				216,179	460,499	5,658,908	2,342,276	8,964,568		44,404,441
**M7**	151,476	1,610,485											1,761,961
**M8**	686,710	509,105							9,795,285	3,463,191			14,454,291
**M9**	5,499,380	4,564,759	247,245	3,396,211	10,439,099	1,349,085	621,654	17,082,221	23,756,273	15,351,670	7,796,211		90,103,808
**M10**	25,071,251												25,071,251
**M11**	6,584,321												6,584,321
**M12**	7,864,503												7,864,503
**M13**	5,673,582	927,152			1,104,655	1,569,801	126,725	2,884,615	10,255,235		861,731		23,403,496
**M14**	4,700,498								1,594,595	2,186,093			8,481,186
**M15**	122,469,048	2,086,502		752,082		1,976,,058			9,533,756	34,065,738			170,883,184
**M16**	10,029,179												10,029,179
**M17**	14,873,427								3,964,569				18,837,996
**M18**	131,377,697	2,775,395				2,221,834		1,382,313	6,051,761		868,285		144,677,285
**M19**	45,423,120	394,065								8,102,455	562,370		54,482,010
**M20**	50,669,383					447,658			1,165,195	34,649,284			86,931,520
**M21**	50,269,416	885,941						128,120	905,385				52,188,862
**M22**	162,025,962	861,129											162,887,091
**M23**	29,484,814												29,484,814
**M24**	180,532,696	352,905						685,128			1,649,227		184,835,243
**M25**	896,803,169	4,472,844				29,155,815		486,612	21,253,177	297,909,967			1,250,081,584
**M26**	48,856,108	1,376,742									598,621		50,831,471
**M27**	790,013,598								2,541,940	18,180,494			810,736,032
**M28**	92,475,813								1,666,710	1,426,867			95,569,390
**M29**	646,804,735	459,452						313,543	12,256,309	1,839,703			661,673,742
**M30**	24,183,728												24,183,728
**M31**	324,786,331								5,151,046				329,937,377
**M32**	390,217,566								818,492				391,036,058
**M33**	46,223,582												46,223,582
**M34**	125,631,723				2,735,164				1,822,325	1,674,281			131,863,493
**M35**	488,223,880				1,363,338				8,760,094	4,522,744			502,870,056
**M36**	33,006,739												33,006,739
**M37**	23,418,337												23,418,337
**M38**	46,101,388												46,101,388
**M39**	27,487,975												27,487,975
**M40**	20,088,773												20,088,773
**M41**	27,408,608												27,408,608
**M42**	12,435,301												12,435,301
**M43**	77,259,589												77,259,589
**M44**	284,733,513												284,733,513
**M45**	44,372,830												44,372,830
**M46**	89,188,731												89,188,731
**M47**	108,520,771												108,520,771
**M48**	30986,855												30,986,855
**M49**	34,753,952												34,753,952
**M50**	53,158,418												53,158,418
**M51**								15,908,470					15,908,470
**M52**					30,236,800			43,804,798			2,369,281		76,410,879
**M53**	463,948,611												463,948,611
**M54**	21,448,937												21,448,937
**M55**	79,454,258												79,454,258
**M56**	79,252,367												79,252,367
**M57**	39,700,636												39,700,636
**M58**	86,848,220												86,848,220
**M59**	17,421,558												17,421,558
**M60**	21,097,294												21,097,294
**M61**	88,863,196												88,863,196
**M62**	38,437,245												38,437,245
**M63**	102,677,796												102,677,796
**M64**	35,880,113												35,880,113
**M65**	23,867,787									1,449,639			25,317,426
**M66**	13,548,270												13,548,270
**M67**	5,583,766												5,583,766
**M68**	5,640,311												5,640,311
**M69**	9,156,984												9,156,984
**M70**	10,312,539												10,312,539
**M71**	13,648,742												13,648,742
**M72**	4,644,757												4,644,757
**M73**	54,507,901												54,507,901
**M74**	77,998,658												77,998,658
**M75**	39,341,209												39,341,209
**M76**	12,303,039												12,303,039
**M77**	7,437,147												7,437,147
**M78**	30,479,610												30,479,610
**M79**	45,861,423												45,861,423
**M80**	18,882,764												18,882,764
**M81**	285,519,682									1,729,889			287,249,571
**M82**	19,376,253												19,376,253
**M83**	36,030,998										26,693,032		62,724,030
**M84**	14,114,954												14,114,954
**M85**	30,984,346							27,085,013					58,069,359
**M86**	50,436,190												50,436,190
**TPA ^2^**	7,540,531,449	125,100,073	1,699,331	4,148,293	50,488,315	37,920,485	1,620,283	115,671,871	147,536,576	449,779,867	53,283,701	0	8,527,780,244
**Sum ^3^**	83	19	2	2	7	7	3	13	21	19	14	0	
**Peak Area (A)**		**A ≥ 10^9^**	**10^9^ >A ≥ 10^8^**		**10^8^ > A ≥ 10^7^**		**10^7^ > A ≥ 10^6^**		**10^6^ > A ≥ 10^5^**		**10^4^ ≤ A < 10^5^**		**A = 0**
**Color**													

^1^ These data are comparable. ^2^ Total peak areas. ^3^ The total number of metabolites detected.

## 3. Discussion

The metabolism of siamenoside I in rats was firstly investigated in the present work. In total, 86 new metabolites of siamenoside I were detected in different biological samples from rats, and nine of them were unambiguously identified by comparison with reference compounds, and the others were tentatively identified by careful interpretation of their LC-MS^n^ data.

### 3.1. The Metabolic Pathways of Siamenoside I in Rats

Based on the structures of the metabolites (**M1**–**M86**), the metabolic pathways of siamenoside I in rats are proposed and shown in Figure 1. From Figure 1, we can find that the metabolic reactions of siamenoside I include deglycosylation, hydroxylation, dehydrogenation, deoxygenation, isomerization, and glycosylation.

Most of the metabolic reactions of siamenoside I are the same to mogroside V [10]. However, there are also some differences. For example, methylation metabolites were not found in the metabolism of siamenoside I, and deoxygenation is found to be a novel metabolic reaction of mogrosides. Furthermore, pentahydroxylation, didehydrogenation are also found as novel metabolic reactions of mogrosides.

Among 86 metabolites, 63 metabolites (**M6**, **M13**, **M20**, **M21**, **M26**, **M27**, **M30**–**M86**) are formed by oxidation reactions such as hydroxylation and dehydrogenation, and 79 metabolites (**M8**–**M86**) are formed by deglycosylation, which indicate that deglycosylation and oxidation (hydroxylation, dehydrogenation) are the major metabolic reactions of siamenoside I.

Astonishingly, four metabolites (**M7**, **M14**, **M22**, and **M23**) are formed by reduction reaction (deoxygenation). The deoxygenation reaction might occur in hexose, not in aglycone, which is inferred from the identification of **M22** (Section 2.2.10.).

Furthermore, 23 metabolites (**M6**, **M7**, **M13**, **M14**, **M22**, **M23**, **M26**-**M28**, **M30**, **M62**-**M65**, **M78**–**M86**) are firstly reported as new metabolites of mogrosides.

These results suggest that siamenoside I has its own metabolism characteristics in comparison with mogroside V.

### 3.2. Distribution of the Metabolites of Siamenoside I in Rats

From Table 2, we could find that total peak areas of all detected compounds (siamenoside I and metabolites) in different rat organs are ranked as follows: intestines (449,779,867) > stomach (147,536,576) > kidneys (115,671,871) > brain (53,283,701) > liver (50,488,315) > spleen (37,920,485) > heart (4,148,293) > lungs (1,620,283). In addition, the total numbers of compounds (siamenoside I and metabolites) detected in different organs are in the order of stomach (22) > intestines (20) > brain (15) > kidneys (14) > liver (7) > spleen (7) > lungs (4) > heart (2). Therefore, siamenoside I and its metabolites are mainly distributed to the intestines, stomach, kidneys, and brain.

We also could find the specific metabolites detected in different biosamples. For example, 50 metabolites (**M10**-**M12**, **M16**, **M23**, **M30**, **M33**, **M36**–**M50**, **M53**-**M64**, **M66**–**M80**, **M82**, **M84**, **M86**) are only detected in feces; **M2** is only detected in urine and the brain; **M7** is only detected in urine and feces; **M51** is only detected in kidneys; **M85** is only detected in kidneys and feces; **M17**, **M31**, **M32** are only detected in stomach and feces; **M65** and **M81** are only detected in intestines and feces; **M83** is only detected in brain and feces. Besides, siamenoside I and 14 metabolites (**M1**–**M6**, **M9**, **M13**, **M18**–**M19**, **M24**, **M26**, **M52**, **M83**) are detected in the brain for the first time.

**M9** (mogroside IIIE) is detected in all biosamples except muscle, indicating that it is the most widely distributed metabolite.

### 3.3. The Proposed in Vivo Process of Siamenoside I in Rats

From Table 2, we could find that **M1**–**M29** seem to be the main metabolites, and most of the other metabolites are only detected in feces. We think the reasons for this result may be: (1) the first pass effect of these metabolites may be very high, *i.e.*, their hepatic extraction ratios are very high, which leads to their very low contents in plasma, organs, and urine samples. As a result, they are not easily detected in these samples; (2) these metabolites are mainly excreted into bile and then to feces, which make their contents in feces high and detectable.

Based on our research results and general metabolic knowledge, we hypothesize that the *in vivo* process of siamenoside I in rats may be as follows.

After oral administration, siamenoside I is degraded into its secondary glycosides (e.g., mogroside III, IIIE, IIIA1, IIE, IIA_2_, IA_1_, IE_1_, *etc.*) and its aglycone (mogrol) or dehydrogenated aglycone (dehydrogenated mogrol) by gastric juice, intestinal juice, intestinal enzymes, or intestinal microflora. Then, mogrol or dehydrogenated mogrol permeate across intestinal mucosa and enter the liver, where they undergo extensive oxidative metabolic reactions to form lots of hydroxylation and/or dehydrogenation metabolites. These polar oxidative metabolites may be largely excreted into the bile and then to the feces, and only a limited amount of them enter general circulation. Besides, some of them may also undergo hepatoenteral circulation and are absorbed into general circulation, and then distributed to different organs, and finally excreted into feces or/and urine.

### 3.4. Bioactivities of the Metabolites of Siamenoside I

On the basis of literature retrieval, eight metabolites (**M3**, mogroside IVA; **M4**, mogroside IVE; **M8**, mogroside III; **M15**, mogroside IIE; **M24**, mogroside IA_1_; **M25**, mogroside IE_1_; **M29**, mogrol; **M9**, mogroside IIIE) of siamenoside I can be regarded as bioactive metabolites.

Among them, seven metabolites (**M3**, **M4**, **M8**, **M15**, **M24**, **M25**, **M29**) are able to inhibit the induction of Epstein-Barr virus early antigen by 12-*O*-tetradecanoylphorbol-13-acetate in Raji cells [5]. **M4** and **M9** can inhibit maltase [7]. Hence, they might contribute to the bioactivities of siamenoside I.

## 4. Materials and Methods

### 4.1. Chemicals and Reagents

Siamenoside I, mogroside III, mogroside IIIE, mogroside IVE, mogroside V, mogroside IIE, 11-oxomogroside IIE, and mogrol (all purities >98%, determined by HPLC-DAD-ELSD) were isolated from the dried fruits of *Siraitia grosvenorii* and the 50% mogroside V enzymatic hydrolysate by the authors [12,13], and their structures were confirmed by spectral data (UV, IR, NMR and MS). Mogroside IVA, mogroside IIIA_1_ and mogroside IIA_2_ (all purities >98%, determined by HPLC-DAD-ELSD) were purchased from Chengdu MUST Bio-technology Co., Ltd. (Chengdu, Sichuan, China).

Ultra-pure water was prepared by a Millipore Milli-Q Integral 3 Ultrapure Water System (Billerica, MA, USA). Acetonitrile (HPLC grade) was bought from Fisher Chemicals Co. (Fairlawn, NJ, USA) and formic acid (HPLC grade) was purchased from Mreda Technology Inc. (Beijing, China). 

### 4.2. Animals

Sprague-Dawley (SD) rats (male, 210 ± 20 g) were bought from the Experimental Animal Center of Peking University Health Science Center (Beijing, China). They were handled in agreement with the Guide for the Care and Use of Laboratory Animals of the US National Institutes of Health. All animal experiments were approved by the Biomedical Ethical Committee of Peking University (Approval No. LA2011-058).

### 4.3. Instruments

A Shimadzu LCMS-IT-TOF instrument was used to perform HPLC-ESI-IT-TOF-MS^n^ analysis, which consists of a CBM-20A system controller, a DGU-20A_3_ degasser, two LC-20AD pumps, an SIL-20AC autosampler, a CTO-20A column oven, an SPD-M20A photodiode array (PDA) detector, an ESI ion source, and a hybrid IT-TOF mass spectrometer (Shimadzu, Kyoto, Japan). 

### 4.4. Animal Experiments and Sample Collection

Eight rats were divided into two groups: two were blank group and the others were test group. Each rat was put into a clean metabolic cage (Suzhou Fengshi Laboratory Animal Equipment Co., Suzhou, Jiangsu, China) and given food and water *ad libitum*.

Because the research aim is to find the general/average differences between test group rats (drug-containing sample) and blank group rats (blank sample), the individual differences among the same group rats are not taken into consideration. Accordingly, all of the biosamples from each group were combined into one sample which was more representative than individual samples in the following sample collection processes.

The animal experiment lasted six days. The whole urine and feces of days 1–2 were collected as blank urine and feces samples, respectively. On days 3–5, the rats of test group were orally administrated with siamenoside I [50 mg/kg body weight, in normal saline (NS) solution] at 9:00, and all 72-h urine and feces were collected as drug-containing urine and feces samples, respectively. The rats of blank group were orally administrated with the same volume of NS. On day 6 at 9:00, the test and the blank group were treated with siamenoside I and NS again, respectively. After 1 h, blood sample was collected into a vacuum tube with sodium citrate as anticoagulant (Hebei Xinle Technology Co., Ltd., Shijiazhuang, Hebei, China) from rat heart under anesthesia. Then, the organs (heart, liver, spleen, lung, kidneys, stomach, small intestine, brain) and skeletal muscles of rats were collected and washed with NS, separately. All samples were kept at −80 °C before further pretreatment.

### 4.5. Sample Preparation

#### 4.5.1. Blood Samples

The blood samples were centrifuged at 3000 rpm for 20 min at 4 °C, and the supernatant plasma samples were collected. Afterward, 8 mL methanol was added to 2 mL of plasma sample, and then was mixed and centrifuged at 9000 rpm for 30 min. The supernatant was collected and evaporated to dryness at 55 °C by a rotatory evaporator. The 15 mg residue was reconstituted in 1.00 mL methanol, filtrated through 0.45 μm filter membrane, and stored at 4 °C before analysis.

#### 4.5.2. Urine Samples

Urine samples were filtered and then evaporated to dryness under vacuum at 55 °C by a rotatory evaporator. Subsequently, the residue was ultrasonically extracted with 10 mL methanol for 30 min, and the extract was then centrifuged at 9000 rpm for 30 min. The supernatant was transferred to another tube and evaporated to dryness at 55 °C by a rotatory evaporator. Next, a 100 mg residue was dissolved in 1.00 mL methanol, filtered through 0.45 μm membranes, and stored at 4 °C before analysis.

#### 4.5.3. Feces Samples

Feces samples were dried at 55 °C and pulverized. Subsequently, the 10 g powder was ultrasonically extracted with 50 mL methanol for 30 min for three times, and the three supernatants were combined and evaporated to dryness. Next, the residue was mixed with 20 mL methanol and centrifuged at 9000 rpm for 30 min. The supernatant was evaporated to dryness again at 55 °C and the residue was collected. Then, 15 mg of residue was dissolved in 1.00 mL methanol, 0.45 μm membranes, and stored at 4 °C before analysis.

#### 4.5.4. Organ and Skeletal Muscle Samples

Each organ was weighed, minced, and homogenized in 4 times volume (mL/g) of 4 °C NS by a homogenizer (Ultra-Turrax T8, Ika-Werke, Gmbh & Co. KG, Staufen, Germany). Then, 10 mL homogenate was mixed with 9 times volume (mL/mL) of acetonitrile, ultrasonically treated for 30 min, and centrifuged at 12,000 rpm for 30 min at 4 °C. Afterward, the supernatant was collected and evaporated to dryness at 50 °C. The residue was dissolved in 1.00 mL methanol, filtered through 0.45 μm filter membrane, and stored at 4 °C before analysis. The skeletal muscle samples were treated by the same method.

### 4.6. LC-MS^n^ Conditions

The column used was Inertsil ODS-3 C_18_ (250 mm × 4.6 mm, 5 μm) (Shimadzu, Kotyo, Japan) protected with a Phenomenex Security Guard column (C_18_, 4 mm × 3.0 mm, 5.0 μm) (Phenomenex, Torrance, CA). The column temperature was 35 °C. The injection volume of all samples was 20 μL. The mobile phases were water-formic acid (100:0.1, *v*/*v*) (A) and acetonitrile (B).The flow rate was 1.0000 mL/min. A gradient elution program was used: 0.01–10.00 min, 10%–18% B; 10.00–20.00 min, 18%–28% B; 20.00–35.00 min, 28%–45% B; 35.00–60.00 min, 45%–90% B; 60.00–70.00 min, 90%–100% B; 70.00–80.00 min, 100% B. The UV spectrum was recorded from 195 nm to 400 nm.

The parameters of the ESI-IT-TOF-MS^n^ instrument were: (1) flow rate: 0.2000 mL/min (split from HPLC effluent); (2) positive ion (PI) and negative ion (NI) alternate detection; (3) mass range: MS, *m*/*z* 300–2000; MS^2^ and MS^3^, *m*/*z* 50–2000; (4) temperature of heat block and curved desolvation line (CDL): 250 °C; (5) nebulizing nitrogen gas flow: 1.5 L/min; interface voltage: (+), 4.5 kV; (−), −3.5 kV; detector voltage: 1.70 kV; (6) ion accumulation time: MS, 30 ms; MS^2^ and MS^3^, 20 ms; relative collision-induced dissociation (CID) energy: 50%; (7) data-dependent MS^2^ and MS^3^ fragmentation; (8) All data were recorded and analyzed by LCMS solution Version 3.60, Formula Predictor Version 1.01, and Accurate Mass Calculator (Shimadzu, Kyoto, Japan). The mass range of 50–3000 Da was calibrated by a trifluoroacetic acid sodium solution (2.5 mM).

### 4.7. Strategy for Profiling and Identification of the Metabolites of Siamenoside I in Biosamples

The strategy [14] previously proposed by the authors was used to find and identify the metabolites of siamenoside I in the present study. In short, the base peak chromatograms (BPCs) of drug-containing samples and blank samples were thoroughly analyzed and compared. Meanwhile, the possible metabolites predicted by general metabolism rules were also screened and confirmed by comparing their corresponding extracted ion chromatograms (EICs). The metabolites were identified by comparison of their retention times and MS^n^ data with those of reference compounds, or tentatively identified by interpretation of their MS^n^ data.

### 4.8. Preliminary Evaluation of the Relative Contents of Siamenoside I and Its Metabolites in Biosamples

To preliminarily estimate the relative contents of siamenoside I and its metabolites in biosamples, the peak area of each metabolite calculated from its NI EIC was used. In the present study, only the data of different organ samples were comparable, since they were prepared and analyzed by the same method.

## 5. Conclusions

The metabolism of siamenoside I in rats was studied for the first time. In total, 86 new metabolites were detected. Nine of them were unambiguously identified by comparison with reference compounds, and the other 77 were tentatively identified by HPLC-ESI-IT-TOF-MS^n^ technique. The metabolic pathways of siamenoside I in rats were proposed based on the structures of metabolites. The metabolic reactions of siamenoside I were found to be deglycosylation, hydroxylation, dehydrogenation, deoxygenation, isomerization, and glycosylation, among which deoxygenation, pentahydroxylation, and didehydrogenation were novel metabolic reactions of mogrosides. In addition, 23 metabolites were new metabolites of mogrosides. The distributions of siamenoside I and its 86 metabolites in rat organs were firstly reported, and they were mainly distributed to intestines, the stomach, kidneys, and the brain. Mogroside IIIE was the most widely distributed metabolite. Eight metabolites had bioactivities, indicating that they might contribute to the bioactivities of siamenoside I. These findings not only provide valuable information on the metabolism and disposition of siamenoside I and mogrosides in rats but also provide useful information on the chemical basis of the pharmacological effects of siamenoside I and mogrosides *in vivo*.

## Supplementary Materials

The following are available online at www.mdpi.com/1420-3049/21/2/176, Figures S1–S25: EICs of 86 metabolites in drug-containing sample and blank sample, Table S1: Retention time (t_R_), LC-ESI-IT-TOF-MS^n^ data, molecular formula, and identification of siamenoside I and its metabolites in different biosamples.

## Abbreviations

The following abbreviations are used in this manuscript:

EBV-EA: Epstein-Barr virus early antigen
TPA: 12-*O*-tetradecanoylphorbol-13-acetate
DMBA: 9,10-Dimethyl-1,2-benzanthracene
HPLC-ESI-IT-TOF-MS^n^: High-performance liquid chromatography-electrospray ionization-ion trap-time of flight-multistage mass spectrometry
Glc: Glucosyl group
NI: Negative ion
DAD-ELSD: Diode array detector coupled with evaporative light scattering detector
NS: Normal saline
BPC: Base peak chromatogram
EIC: Extracted ion chromatogram
