# Supplementary material for: Metabolites of Siamenoside I and Their Distributions in Rats
Source: Molecules. 2016 Jan 30;21(2):176. doi: 10.3390/molecules21020176 (PMC6274126; doi:10.3390/molecules21020176)
Supplement: Supplementary file 1 [file molecules-21-00176-s001.docx]

**Supplementary Materials: Metabolites of Siamenoside I and Their Distributions in Rats**

Xue-Rong Yang, Feng Xu, Dian-Peng Li Feng-Lai Lu, Guang-Xue Liu, Lei Wang, Ming-Ying Shang, Yong-Lin Huang and Shao-Qing Cai

**Table S1.** Retention time (t_R_), LC-ESI-IT-TOF-MS^n^ data, molecular formula, and identification of siamenoside I and its metabolites in different biosamples.

| **No.** | **Sample** | **t_R_ (min)** | **Measured Mass (Da) of [M + X]^+^** | **Predicated Mass (Da) of [M + X]^+^** | **Error (ppm)** | **Characteristic Positive Fragment Ions (Relative Abundance)** | **Measured Mass (Da) of [M + X]^−^** | **Predicated Mass (Da) of [M + X]^−^** | **Error (ppm)** | **Characteristic Negative Fragment Ions (Relative Abundance)** | **DBE** | **Molecular Formula** | **Exact Mass** | **Identification** |
| --- | --- | --- | --- | --- | --- | --- | --- | --- | --- | --- | --- | --- | --- | --- |
| **M0**^a^ | feces | 24.770  −25.078 |  |  |  |  | 1169.5959 [M + HCOOH − H]^−^ | 1169.5961 | 0.17 | MS^2^: 961.5303(35.24), C_48_H_81_O_19_; 799.4797(100), C_42_H_71_O_14_; 475.3799(4.14), C_30_H_51_O_4_. MS^3^(799.47969): 637.313(100), C_36_H_61_O_9_; 475.3710(10.43), C_30_H_51_O_4_. | 9 | C_54_H_92_O_24_ | 1124.5979 | siamenoside I  |
| **M1** | urine | 25.500 −25.817 | 1287.6523 [M + H]^+^ 23.625 | 1287.6580 | −4.43 | − | 1285.6444 [M − H]^−^ | 1285.6434 | 0.78 | MS^2^: 1123.5827(47.10), C_54_H_91_O_24_; 961.5276(63.10), C_48_H_81_O_19_; 799.4830(100), C_42_H_71_O_14_; 637.4258(43.58). MS^3^(799.4830): 637.4086(100), C_36_H_61_O_9_; 475.3813(100), C_30_H_51_O_4_. | 10 | C_60_H_102_O_29_ | 1286.6507 | mogroside V isomer |

**Table S1.** *Cont*.

| **No.** | **Sample** | **t_R_ (min)** | **Measured Mass (Da) of [M + X]^+^** | **Predicated Mass (Da) of [M + X]^+^** | **Error (ppm)** | **Characteristic Positive Fragment Ions (Relative Abundance)** | **Measured mass (Da) of [M + X]^−^** | **Predicated Mass (Da) of [M + X]^−^** | **Error (ppm)** | **Characteristic Negative Fragment Ions (Relative Abundance)** | **DBE** | **Molecular Formula** | **Exact Mass** | **Identification** |
| --- | --- | --- | --- | --- | --- | --- | --- | --- | --- | --- | --- | --- | --- | --- |
| **M2** | urine | 25.817 −26.300 | 1287.6523 [M + H]^+^ | 1287.6580 | −4.43 | − | 1285.6441 [M − H]^−^ | 1285.6434 | 0.54 | MS^2^: 1123.5785(39.85), C_54_H_91_O_24_; 961.5342(83.96), C_48_H_81_O_19_; 799.4830(100), C_42_H_71_O_14_; 637.4284(63.14), C_36_H_61_O_9_; 475.3780(4.64). C_36_H_61_O_9_. MS^3^(799.4821): 637.4294(100), C_36_H_61_O_9_; | 10 | C_60_H_102_O_29_ | 1286.6507 | mogroside V isomer |
| **M3** | feces | 25.285 −25.650 |  |  |  |  | 1169.5905 [M + HCOOH − H]^−^ | 1169.5961 | −0.09 | MS^2^: 1123.5708(100), C_54_H_91_O_24_. | 9 | C_54_H_92_O_24_ | 1124.5979 | mogroside IVA  |
| **M4** | feces | 25.650 −25.933 |  |  |  |  | 1169.5923 [M + HCOOH − H]^−^ | 1169.5961 | −3.25 | MS^2^: 961.5131(1.81), C_48_H_81_O_24._ MS^3^(1123.5814): 961.5241(34.61), C_48_H_81_O_19;_ 799.4830(100), C_42_H_71_O_14_; 637.4301(37.11), C_36_H_61_O_9_; 475.3778(9.18), C_36_H_61_O_9_. | 9 | C_54_H_92_O_24_ | 1124.5979 | mogroside IVE  |
| **M5** | feces | 26.517 −26.950 |  |  |  |  | 1169.5950 [M + HCOOH − H]^−^ | 1169.5961 | −0.94 | MS^2^(1123.5823): 961.5351(66.23), C_48_H_81_O_19;_ 799.4743(100), C_42_H_71_O_14_; 637.4300(51.45), C_36_H_61_O_9_; 475.3903(8.87), C_36_H_61_O_9_. | 9 | C_54_H_92_O_24_ | 1124.5979 | mogroside IV isomer |

**Table S1.** *Cont*.

| **No.** | **Sample** | **t_R_ (min)** | **Measured Mass (Da) of [M + X]^+^** | **Predicated Mass (Da) of [M + X]^+^** | **Error (ppm)** | **Characteristic Positive Fragment Ions (Relative Abundance)** | **Measured Mass (Da) of [M + X]^−^** | **Predicated Mass (Da) of [M + X]^−^** | **Error (ppm)** | **Characteristic Negative Fragment Ions (Relative Abundance)** | **DBE** | **Molecular Formula** | **Exact Mass** | **Identification** |
| --- | --- | --- | --- | --- | --- | --- | --- | --- | --- | --- | --- | --- | --- | --- |
| **M6** | urine | 25.753 −26.103 |  |  |  |  | 1167.5730 [M + HCOOH − H]^−^ | 1167.5804 | −2.23 | MS^2^: 959.5171(62.40), C_48_H_79_O_19;_ 797.4653(100), C_42_H_69_O_14;_ 635.4113(45.16), C_36_H_59_O_9;_ 473.3605(4.97), C_30_H_49_O_4._ MS^3^(797.4697): 635.4181(100), C_36_H_59_O_9;_ 473.4600(65.78),  C_30_H_49_O_4._ | 10 | C_54_H_90_O_24_ | 1122.5822 | dehydrogenated siamenoside I |
| **M7** | urine | 25.800 −26.117 | − | − | − | − | 1153.5957 [M + HCOOH − H]^−^ 1143.5710 [M + Cl − H]^−^ | 1153.6011 1143.5723 | −4.25 −1.14 | − | 9 | C_54_H_92_O_23_ | 1108.6029 | deoxygenated siamenoside I |
| **M8** | feces | 26.717 −27.050 | − | − | − | − | 1007.5425 [M + HCOOH − H]^−^ 997.5097 [M + Cl − H]^−^ 961.5325 [M − H]^−^ | 1007.5432 997.5144 961.5378 | −0.69 −4.71 −5.51 | − | 8 | C_48_H_82_O_19_ | 962.5450 | mogroside III |
| **M9** | feces | 27.050 −27.633 | − | − | − | − | 1007.5387 [M + HCOOH − H]^−^ 997.5088 [M + Cl − H]^−^ 961.5357 [M − H]^−^ | 1007.5432 997.5144 961.5378 | −4.47 −5.61 −2.18 | MS^2^: 799.4806(100), C_42_H_71_O_14_; 637.4259(65.66), C_36_H_61_O_9_; 475.3758(10.23), C_30_H_51_O_4_. MS^3^(799.4805): 637.4278(100), C_36_H_61_O_9_; 475.3774(57.24), C_30_H_51_O_4_. | 8 | C_48_H_82_O_19_ | 962.5450 | mogroside IIIE  |

**Table S1.** *Cont*.

| **No.** | **Sample** | **t_R_ (min)** | **Measured Mass (Da) of [M + X]^+^** | **Predicated Mass (Da) of [M + X]^+^** | **Error (ppm)** | **Characteristic Positive Fragment Ions (Relative Abundance)** | **Measured Mass (Da) of [M + X]^−^** | **Predicated Mass (Da) of [M + X]^−^** | **Error (ppm)** | **Characteristic Negative Fragment Ions (Relative Abundance)** | **DBE** | **Molecular Formula** | **Exact Mass** | **Identification** |
| --- | --- | --- | --- | --- | --- | --- | --- | --- | --- | --- | --- | --- | --- | --- |
| **M10** | feces | 30.017 −30.400 | 963.5469 | 963.5523 | −5.60 | − | 1007.5376[M + HCOOH − H]^−^ | 1007.5432 | −5.56 | MS^2^: 961.5284(100), C_48_H_81_O_19_. | 8 | C_48_H_82_O_19_ | 962.5450 | mogroside IIIA_1_  |
| **M11** | feces | 30.400 −30.767 | − | − | − | − | 1007.5395 [M + HCOOH − H]^−^ 997.5069 [M + Cl − H]^−^ 961.5353 [M − H]^−^ | 1007.5432 997.5144 961.5378 | −3.67 −7.51 −2.60 | - | 8 | C_48_H_82_O_19_ | 962.5450 | mogroside III isomer |
| **M12** | feces | 30.767 −31.383 | − | − | − | − | 1007.5385 [M + HCOOH − H]^−^ 997.5174 [M+Cl−H]^−^ | 1007.5432 997.5144 | −4.66 −3.01 | − | 8 | C_48_H_82_O_19_ | 962.5450 | mogroside III isomer |
| **M13** | feces | 26.800 −27.117 | − | − | − | − | 1005.5269 [M + HCOOH − H]^−^ | 1005.5276 | 0.70 | MS^2^: 797.4629(100), C_42_H_69_O_14;_ 635.4090(49.23), C_36_H_59_O_9;_ 473.3623(8.55), C_30_H_49_O_4._ MS^3^(797.46297): 635.3972(100), C_36_H_59_O_9;_ 473.3654(54.93), C_30_H_49_O_4._ | 9 | C_48_H_80_O_19_ | 960.5294 | dehydrogenated mogroside III isomer |
| **M14** | feces | 27.383 −28.000 |  |  |  |  | 991.5441 [M + HCOOH − H]^−^ 981.5224 [M + Cl − H]^−^ 945.5431 [M − H]^−^ | 991.5483 981.5195 945.5428 | −4.24 2.95 0.32 | − |  | C_48_H_82_O_18_ | 946.5501 | deoxygenated mogroside III isomer |

**Table S1.** *Cont*.

| **No.** | **Sample** | **t_R_ (min)** | **Measured Mass (Da) of [M + X]^+^** | **Predicated Mass (Da) of [M + X]^+^** | **Error (ppm)** | **Characteristic Positive Fragment Ions (Relative Abundance)** | **Measured Mass (Da) of [M + X]^−^** | **Predicated Mass (Da) of [M + X]^−^** | **Error (ppm)** | **Characteristic Negative Fragment Ions (Relative Abundance)** | **DBE** | **Molecular Formula** | **Exact Mass** | **Identification** |
| --- | --- | --- | --- | --- | --- | --- | --- | --- | --- | --- | --- | --- | --- | --- |
| **M15** | feces | 29.183 −29.717 |  |  |  | − | 845.4881 [M + HCOOH − H]^−^ 799.4816 [M−H]^−^ | 845.4904 799.4849 | −2.72 2.13 | MS^2^: 799.4784(100), C_42_H_71_O_14_; 637.4140(6.89), C_36_H_61_O_9_. MS^3^(799.4781): 473.3757(100), C_30_H_49_O_4._ | 7 | C_42_H_72_O_14_ | 800.4922 | mogroside IIE  |
| **M16** | feces | 30.467 −30.883 | − | − | − | − | 845.4944 [M + HCOOH − H]^−^ 799.485 [M − H]^−^ 31.683 | 845.4904 799.4849 | 4.73 0.25 | − | 7 | C_42_H_72_O_14_ | 800.4922 | mogroside II isomer |
| **M17** | feces | 31.433 −32.333 | − | − | − | − | 845.4910 [M + HCOOH − H]^−^ 799.4816 [M − H]^−^ 29.305 | 845.4904 799.4849 | 0.71 −2.75 | − | 7 | C_42_H_72_O_14_ | 800.4922 | mogroside IIA_2_  |
| **M18** | feces | 33.000 −33.500 | − | − | − | − | 845.4884 [M+HCOOH − H]^−^ 799.4858 [M − H]^−^ 33.085 | 845.4904 799.4849 | −2.37 −1.13 | MS^2^: 637.4295(75.39), C_36_H_61_O_9_; 475.3760(100), C_30_H_51_O_4_; 459.3444(30.45), C_29_H_47_O_4_. MS^3^(475.3760): 459.3462(100), C_29_H_47_O_4_; 429.3290(95.70), C_28_H_45_O_3_; 415.3167(33.94), C_27_H_43_O_3_. | 7 | C_42_H_72_O_14_ | 800.4922 | mogroside II isomer |

**Table S1.** *Cont*.

| **No.** | **Sample** | **t_R_ (min)** | **Measured Mass (Da) of [M + X]^+^** | **Predicated Mass (Da) of [M + X]^+^** | **Error (ppm)** | **Characteristic Positive Fragment Ions (Relative Abundance)** | **Measured Mass (Da) of [M + X]^−^** | **Predicated Mass (Da) of [M + X]^−^** | **Error (ppm)** | **Characteristic Negative Fragment Ions (Relative Abundance)** | **DBE** | **Molecular Formula** | **Exact Mass** | **Identification** |
| --- | --- | --- | --- | --- | --- | --- | --- | --- | --- | --- | --- | --- | --- | --- |
| **M19** | feces | 33.500 −34.100 | 823.4816 [M + Na]^+^ 33.743 | 823.4814 | 0.24 | − | 845.4918 [M + HCOOH − H]^−^ 799.4864 [M − H]^−^ 33.620 | 845.4904 799.4849 | 1.66 1.88 | − | 7 | C_42_H_72_O_14_ | 800.4922 | mogroside II isomer |
| **M20** | feces | 29.847 −30.278 | 821.4603 [M + Na]^+^ | 821.4658 | −6.70 | − | 843.4737 [M+HCOOH−H]^−^ 29.853 | 843.4748 | −1.30 | MS^2^: 635.4108(100), C_36_H_59_O_9_; 473.3591(28.370), C_30_H_49_O_4_. | 8 | C_42_H_70_O_14_ | 798.4766 | 11-oxomogroside IIE  |
| **M21** | feces | 33.350 −33.917 | − | − | − | − | 843.4726 [M + HCOOH − H]^−^ | 843.4748 | −2.61 | MS^2^: 797.4643(100), C_42_H_69_O_14_. MS^3^(797.4643): 473.3506(100), C_30_H_49_O_4_. | 8 | C_42_H_70_O_14_ | 798.4766 | dehydrogenated mogroside II isomer |
| **M22** | feces | 33.700 −34.517 | − | − | − | − | 819.4647 [M + Cl − H]^−^ 829.4946 [M + HCOOH − H]^−^ | 819.4667 829.4955 | −2.44 −1.08 | MS^2^: 783.4833(100), C_42_H_71_O_13;_ 637.4252(49.91), C_36_H_61_O_9_; 475.3742(7.12), C_30_H_51_O_4_. MS^3^(783.4830): 475.3558(100), C_30_H_51_O_4_; | 7 | C_42_H_72_O_13_ | 784.4973 | deoxygenated mogroside II isomer |
| **M23** | feces | 34.567 −35.183 | 783.4785 | 783.4737 | 6.13 | − | 827.4780 [M+HCOOH−H]^−^ 817.4510 [M+Cl−H]^−^ 781.4708 [M−H]^−^ | 827.4798 817.4510 781.4744 | −2.18 0 −4.61 | − | 8 | C_42_H_70_O_13_ | 782.4816 | dehydrogenated deoxygenated mogroside II isomer |

**Table S1.** *Cont*.

| **No.** | **Sample** | **t_R_ (min)** | **Measured Mass (Da) of [M + X]^+^** | **Predicated Mass (Da) of [M + X]^+^** | **Error (ppm)** | **Characteristic Positive Fragment Ions (Relative Abundance)** | **Measured Mass (Da) of [M + X]^−^** | **Predicated Mass (Da) of [M + X]^−^** | **Error (ppm)** | **Characteristic Negative Fragment Ions (Relative Abundance)** | **DBE** | **Molecular Formula** | **Exact Mass** | **Identification** |
| --- | --- | --- | --- | --- | --- | --- | --- | --- | --- | --- | --- | --- | --- | --- |
| **M24** | feces | 34.767 −35.433 | − | − | − | − | 683.4348 [M + HCOOH − H]^−^ 637.4303 [M − H]^−^ | 683.4376 637.4321 | −1.76 −2.82 | MS^2^: 637.4278(100), C_36_H_61_O_9_; 475.3661(0.56), C_30_H_51_O_4_. MS^3^(637.4279): 475.3755(100), C_30_H_51_O_4_; 473.3640(18.71), C_30_H_49_O_4_; 459.3469(21.35), C_29_H_47_O_4_. | 6 | C_36_H_62_O_9_ | 638.4394 | mogroside IA_1_  |
| **M25** | feces | 36.950 −37.983 | − | − | −5.59 | − | 683.4366 [M + HCOOH − H]^−^ | 683.4376 | 1.46 | MS^2^: 637.4266(100), C_36_H_61_O_9_; 475.3774(28.26), C_30_H_51_O_4_. MS^3^(637.4264): 475.3746(79.82), C_30_H_51_O_4_; 473.3642(14.44), C_30_H_49_O_4_; 459.3393(12.85), C_29_H_47_O_4_; | 6 | C_36_H_62_O_9_ | 638.4394 | mogroside IE_1_  |
| **M26** | feces | 35.883 −36.500 | − | − | − | − | 681.4196 [M + HCOOH − H]^−^ 635.4176 [M − H]^−^ | 681.4219 635.4165 | −3.38 1.73 | MS^2^: 635.4114(100), C_36_H_59_O_9;_ MS^3^(635.4116): 471.3587(100), C_30_H_47_O_4._ 473.3654(75.24), C_30_H_49_O_4._ | 7 | C_36_H_60_O_9_ | 636.4237 | dehydrogenated mogroside I isomer |

**Table S1.** *Cont*.

| **No.** | **Sample** | **t_R_ (min)** | **Measured Mass (Da) of [M + X]^+^** | **Predicated Mass (Da) of [M + X]^+^** | **Error (ppm)** | **Characteristic Positive Fragment Ions (Relative Abundance)** | **Measured Mass (Da) of [M + X]^−^** | **Predicated Mass (Da) of [M + X]^−^** | **Error (ppm)** | **Characteristic Negative Fragment Ions (Relative Abundance)** | **DBE** | **Molecular Formula** | **Exact Mass** | **Identification** |
| --- | --- | --- | --- | --- | --- | --- | --- | --- | --- | --- | --- | --- | --- | --- |
| **M27** | feces | 38.433 −39.400 | − | − | − |  | 681.4215 [M + HCOOH − H]^−^ | 681.4219 | −0.59 | MS^2^: 635.4155(100), C_36_H_59_O_9_; 473.3588(28.81), C_30_H_49_O_4._ MS^3^(635.4156): 473.3757(100), C_30_H_49_O_4._ | 7 | C_36_H_60_O_9_ | 636.4237 | dehydrogenated mogroside I isomer |
| **M28** | feces | 45.683 −46.233 | − | − | − | − | 521.3834 [M+HCOOH−H]^−^ 511.3539 [M+Cl−H]^−^ | 521.3848 511.3539 | −2.69 −4.11 | − | 5 | C_30_H_52_O_4_ | 476.3866 | mogrol isomer |
| **M29** | feces | 46.233 −47.133 |  | − | − | − | 521.3838 [M + HCOOH − H]^−^ 511.3554 [M + Cl − H]^−^ | 521.3848 511.3560 | −1.92 −1.17 | − | 5 | C_30_H_52_O_4_ | 476.3866 | mogrol  |
| **M30** | feces | 52.000 −52.667 | − | − | − | − | 519.3676 [M + HCOOH − H]^−^ 509.3385 [M + Cl − H]^−^ | 519.3691 509.3403 | −2.89 −3.53 | MS^2^: 459.3189(100), C_29_H_47_O_4_; | 6 | C_30_H_50_O_4_ | 474.3709 | dehydrogenated mogrol |
| **M31** | feces | 52.667 −53.383 | − | − | − | − | 519.3687 [M + HCOOH − H]^−^ | 519.3691 | −0.77 | MS^2^: 459.3189(100), C_29_H_47_O_4_; 407.2888(11.12), C_24_H_35_O_5_; 433.3405(6.67), C_27_H_45_O_4_. MS^3^(459.3087): 407.2888(100), C_27_H_45_O_4_. | 6 | C_30_H_50_O_4_ | 474.3709 | dehydrogenated mogrol |

**Table S1.** *Cont*.

| **No.** | **Sample** | **t_R_ (min)** | **Measured Mass (Da) of [M + X]^+^** | **Predicated Mass (Da) of [M + X]^+^** | **Error (ppm)** | **Characteristic Positive Fragment Ions (Relative Abundance)** | **Measured Mass (Da) of [M + X]^−^** | **Predicated Mass (Da) of [M + X]^−^** | **Error (ppm)** | **Characteristic Negative Fragment Ions (Relative Abundance)** | **DBE** | **Molecular Formula** | **Exact Mass** | **Identification** |
| --- | --- | --- | --- | --- | --- | --- | --- | --- | --- | --- | --- | --- | --- | --- |
| **M32** | feces | 22.600 −23.433 | − | − | −9.60 | − | 553.3721 [M + HCOOH − H]^−^ | 553.3746 | −4.52 | MS^2^: 507.3653(100), C_30_H_51_O_6_; 489.3318(6.75), C_30_H_49_O_5_; 433.3011(6.75), C_27_H_45_O_4_; 349.2406(9.15), C_21_H_33_O_4_. | 5 | C_30_H_52_O_6_ | 508.3764 | dihydroxylated mogrol |
| **M33** | feces | 26.300 −26.767 | − | − | − | − | 553.3699 [M + HCOOH − H]^−^ | 553.3746 | −8.49 | MS^2^: 507.3569(100), C_30_H_51_O_6_; 399.2257(8.52), C_25_H_35_O_4_. MS^3^(507.3568): 211.1720(100), C_13_H_23_O_2._ | 5 | C_30_H_52_O_6_ | 508.3764 | dihydroxylated mogrol |
| **M34** | feces | 27.183 −27.750 | − | − | − | − | 553.3717 [M + HCOOH − H]^−^ | 553.3746 | −5.24 | MS^2^: 453.2782(100), C_29_H_41_O_4_. | 5 | C_30_H_52_O_6_ | 508.3764 | dihydroxylated mogrol |
| **M35** | feces | 27.967 −28.633 | 531.3606 [M+Na]^+^ | 531.3656 | −9.41 | MS^2^: 513.3359 (78.46), C_30_H_51_O_5_. | 553.3740 [M + HCOOH − H]^−^ | 553.3746 | −1.08 | MS^2^: 399.2155(44.43), C_25_H_35_O_4_. MS^3^(399.2155): 355.2123, C_20_H_35_O_5_. | 5 | C_30_H_52_O_6_ | 508.3764 | dihydroxylated mogrol |
| **M36** | feces | 26.117 −26.767 | 507.3610 [M+H]^+^ | 507.3680 | −13.80 | − | 551.3559 [M + HCOOH − H]^−^ | 551.3589 | −5.40 | − | 6 | C_30_H_50_O_6_ | 506.3607 | dehydrogenated dihydroxylated mogrol |
| **M37** | feces | 26.767 −27.367 | − | − | − | − | 551.3557 [M + HCOOH − H]^−^ | 551.3589 | −5.80 | MS^2^: 505.3531(100), C_30_H_49_O_6_; 435.2746(28.73), C_25_H_39_O_6_. | 6 | C_30_H_50_O_6_ | 506.3607 | dehydrogenated dihydroxylated mogrol |
| **M38** | feces | 29.183 −29.683 | − | − | − | − | 551.3566 [M + HCOOH − H]^−^ | 551.3589 | −4.17 | MS^2^: 447.2498(100), C_26_H_39_O_6_. | 6 | C_30_H_50_O_6_ | 506.3607 | dehydrogenated dihydroxylated mogrol |

**Table S1.** *Cont*.

| **No.** | **Sample** | **t_R_ (min)** | **Measured Mass (Da) of [M + X]^+^** | **Predicated Mass (Da) of [M + X]^+^** | **Error (ppm)** | **Characteristic Positive Fragment Ions (Relative Abundance)** | **Measured Mass (Da) of [M + X]^−^** | **Predicated Mass (Da) of [M + X]^−^** | **Error (ppm)** | **Characteristic Negative Fragment Ions (Relative Abundance)** | **DBE** | **Molecular Formula** | **Exact Mass** | **Identification** |
| --- | --- | --- | --- | --- | --- | --- | --- | --- | --- | --- | --- | --- | --- | --- |
| **M39** | feces | 30.283 −31.250 |  |  |  | − | 551.3558 [M + HCOOH − H]^−^ | 551.3589 | −5.62 | MS^2^: 505.3501(100), C_30_H_49_O_6_. | 6 | C_30_H_50_O_6_ | 506.3607 | dehydrogenated dihydroxylated mogrol |
| **M40** | feces | 31.250 −31.833 | − | − | − | − | 551.3561 [[M + HCOOH − H]^−^ | 551.3589 | −5.04 | MS^2^: 504.3394(100), C_30_H_48_O_6_. | 6 | C_30_H_50_O_6_ | 506.3607 | dehydrogenated dihydroxylated mogrol |
| **M41** | feces | 32.867 −33.367 | − | − | − | − | 551.3549 [M + HCOOH − H]^−^ | 551.3589 | −7.25 | − | 6 | C_30_H_50_O_6_ | 506.3607 | dehydrogenated dihydroxylated mogrol |
| **M42** | feces | 16.117 −16.500 | − | − | − | − | 569.3655 [M + HCOOH − H]^−^ | 569.3695 | −7.03 | MS^2^: 523.3486(100), C_30_H_51_O_7_; 475.3374(61.86), C_29_H_47_O_5_. | 5 | C_30_H_52_O_7_ | 524.3713 | trihydroxylated mogrol |
| **M43** | feces | 16.500 −17.033 | 547.3553 [M + Na]^+^ 16.950 | 547.3605 | −9.50 | MS^2^: 443.3032 (100), C_26_H_44_O_4_Na. | 569.3671 [M + HCOOH − H]^−^ | 569.3695 | −4.22 | MS^2^: 523.3609(100), C_30_H_51_O_7_; 369.2762(50), C_21_H_37_O_5_. MS^3^(523.3609): 369.2702(100), C_21_H_37_O_5_ | 5 | C_30_H_52_O_7_ | 524.3713 | trihydroxylated mogrol |
| **M44** | feces | 17.033 −17.767 | 547.3572 [M + Na]^+^ | 547.3605 | −6.03 | − | 569.3660 [M + HCOOH − H]^−^ | 569.3695 | −6.15 | MS^2^: 523.3472(100), C_30_H_51_O_7_; 447.2889(60.16), C_26_H_39_O_6_. | 5 | C_30_H_52_O_7_ | 524.3713 | trihydroxylated mogrol |
| **M45** | feces | 17.767 −18.617 | 547.3553 [M + Na]^+^ 18.225 | 547.3605 | −9.50 | MS^2^: 443.3131 (100), C_26_H_44_O_4_Na. | 569.3674 [M + HCOOH − H]^−^ | 569.3695 | −3.69 | MS^2^: 523.33596(100), C_30_H_51_O_7_; 505.3394(3.85), C_30_H_49_O_6._ MS^3^(523.3595): 457.3195(85.39), C_29_H_45_O_4_; 439.3144(85.39), C_29_H_43_O_3_. | 5 | C_30_H_52_O_7_ | 524.3713 | trihydroxylated mogrol |

**Table S1.** *Cont*.

| **No.** | **Sample** | **t_R_ (min)** | **Measured Mass (Da) of [M + X]^+^** | **Predicated Mass (Da) of [M + X]^+^** | **Error (ppm)** | **Characteristic Positive Fragment Ions (Relative Abundance)** | **Measured Mass (Da) of [M + X]^−^** | **Predicated Mass (Da) of [M + X]^−^** | **Error (ppm)** | **Characteristic Negative Fragment Ions (Relative Abundance)** | **DBE** | **Molecular Formula** | **Exact Mass** | **Identification** |
| --- | --- | --- | --- | --- | --- | --- | --- | --- | --- | --- | --- | --- | --- | --- |
| **M46** | feces | 18.617 −19.333 | 547.3561 [M + Na]^+^ 19.027 | 547.3605 | −8.04 | − | 569.3675 [M + HCOOH − H]^−^ | 569.3695 | −3.51 | MS^2^: 523.3614(100), C_30_H_51_O_7_; 505.3486(8.24), C_30_H_50_O_6_; 487.3385(34.10), C_30_H_47_O_5_; 381.2748(21.196), C_22_H_37_O_5_. MS^3^(523.3613): 381.2743(100), C_22_H_37_O_5_^-^. | 5 | C_30_H_52_O_7_ | 524.3713 | trihydroxylated mogrol |
| **M47** | feces | 21.150 −21.650 | − | − | − | − | 569.36 75 [M + HCOOH − H]^−^ | 569.3695 | −3.51 | MS^2^: 523.3698(100), C_30_H_51_O_7;_ 381.2676(100), C_22_H_37_O_5_. | 5 | C_30_H_52_O_7_ | 524.3713 | trihydroxylated mogrol |
| **M48** | feces | 21.650 −21.933 | 547.3561 [M + Na]^+^ 21.783 | 547.3605 | −8.04 | − | 569.3674 [M + HCOOH − H]^−^ | 569.3695 | −3.69 | − | 5 | C_30_H_52_O_7_ | 524.3713 | trihydroxylated mogrol |
| **M49** | feces | 21.933 −22.300 | − | − | − | − | 569.3666 [M + HCOOH − H]^−^ | 569.3695 | −5.09 | − | 5 | C_30_H_52_O_7_ | 524.3713 | trihydroxylated mogrol |
| **M50** | feces | 22.300 −22.700 | − | − | − | − | 569.3655 [[M + HCOOH − H]^−^ | 569.3695 | −7.03 | MS^2^: 523.3617(100), C_30_H_51_O_7_; 505.3501(2), C_30_H_49_O_6._ | 5 | C_30_H_52_O_7_ | 524.3713 | trihydroxylated mogrol |
| **M51** | kidney | 11.233 −11.783 | − | − | − | − | 567.3489 [M+HCOOH−H]^−^ | 567.3539 | −8.80 | − | 6 | C_30_H_50_O_7_ | 522.3557 | dehydrogenated trihydroxylated mogrol |
| **M52** | kidney | 18.233 −18.833 | − | − | − | − | 567.3463 [M + HCOOH − H]^−^ | 567.3539 | −13.4 | − | 6 | C_30_H_50_O_7_ | 522.3557 | dehydrogenated trihydroxylated mogrol |

**Table S1.** *Cont*.

| **No.** | **Sample** | **t_R_ (min)** | **Measured Mass (Da) of [M + X]^+^** | **Predicated Mass (Da) of [M + X]^+^** | **Error (ppm)** | **Characteristic Positive Fragment Ions (Relative Abundance)** | **Measured Mass (Da) of [M + X]^−^** | **Predicated Mass (Da) of [M + X]^−^** | **Error (ppm)** | **Characteristic Negative Fragment Ions (Relative Abundance)** | **DBE** | **Molecular Formula** | **Exact Mass** | **Identification** |
| --- | --- | --- | --- | --- | --- | --- | --- | --- | --- | --- | --- | --- | --- | --- |
| **M53** | feces | 19.650 −20.483 | − | − | − | − | 567.3524 [M + HCOOH − H]^−^ | 567.3539 | −2.64 | MS^2^: 521.3207(59.89), C_30_H_49_O_7_; 503.3364(100), C_30_H_47_O_6_; 487.3207(34.45), C_30_H_47_O_5_; 397.2713(59.98). MS^3^(503.3365): 487.2810(17.40), C_30_H_47_O_5_; 485.3270(18.98), C_30_H_45_O_5_; 397.2753(100), C_26_H_37_O_3_; 355.2569(28.85), C_20_H_35_O_5_. | 6 | C_30_H_50_O_7_ | 522.3557 | dehydrogenated trihydroxylated mogrol |
| **M54** | feces | 20.750 −21.233 | − | − | − | − | 567.3534 [[M + HCOOH − H]^−^ | 567.3539 | −0.88 | MS^2^: 521.3439(46.21), C_30_H_49_O_7_; 505.3453(14.22), C_30_H_49_O_6_; 503.3312(100), C_30_H_47_O_6_; 487.3062(15.89), C_30_H_47_O_5_; 397.2710(59.98), C_26_H_37_O_3_;  MS^3^(503.3310): 485.3180(15.29), C_30_H_45_O_5_^-^; 397.2753(90.85), C_26_H_37_O_3_; 355.2648(100), C_20_H_35_O_5_. | 6 | C_30_H_50_O_7_ | 522.3557 | dehydrogenated trihydroxylated mogrol |

**Table S1.** *Cont*.

| **No.** | **Sample** | **t_R_ (min)** | **Measured Mass (Da) of [M + X]^+^** | **Predicated Mass (Da) of [M + X]^+^** | **Error (ppm)** | **Characteristic Positive Fragment Ions (Relative Abundance)** | **Measured Mass (Da) of [M + X]^−^** | **Predicated Mass (Da) of [M + X]^−^** | **Error (ppm)** | **Characteristic Negative Fragment Ions (Relative Abundance)** | **DBE** | **Molecular Formula** | **Exact Mass** | **Identification** |
| --- | --- | --- | --- | --- | --- | --- | --- | --- | --- | --- | --- | --- | --- | --- |
| **M55** | feces | 21.467 −22.000 | 545.3421 [M + Na]^+^ 20.072 | 545.3449 | −5.13 | − | 567.3494 [M + HCOOH − H]^−^ | 567.3539 | −7.93 | MS^2^: 521.3476(44.06), C_30_H_49_O_7_; 503.3342(24.40), C_30_H_47_O_6_; 371.2569(100), C_22_H_29_O_2_+HCOOH; 419.2780(44.89), C_25_H_39_O_5_. | 6 | C_30_H_50_O_7_ | 522.3557 | dehydrogenated trihydroxylated mogrol |
| **M56** | feces | 22.000 −22.550 | 545.3429 [M + Na]^+^ 21.050 | 545.3449 | −3.67 | − | 567.3512 [M + HCOOH − H]^−^ | 567.3539 | −4.76 | MS^2^: 473.3237(100), C_30_H_49_O_4_; 455.3126(26.28), C_29_H_43_O_4_. MS^3^(473.3236): 455.3168, C_29_H_43_O_4_. | 6 | C_30_H_50_O_7_ | 522.3557 | dehydrogenated trihydroxylated mogrol |
| **M57** | feces | 23.867 −24.300 | 545.3408 [M + Na]^+^ | 545.3449 | −7.52 | − | 567.3507 [M + HCOOH − H]^−^ | 567.3539 | −5.64 | MS^2^: 521.3479(100), C_30_H_49_O_7_; 503.3399(97.10), C_30_H_47_O_6_; 485.3339(27.26), C_30_H_45_O_5_; 397.27.26(25.00), C_26_H_37_O_3_. MS^3^(521.3478): 503.3481(100), C_30_H_47_O_6_; 485.3180(55.05), C_30_H_45_O_5_; 397.260355.05), C_26_H_37_O_3_. | 6 | C_30_H_50_O_7_ | 522.3557 | dehydrogenated trihydroxylated mogrol |
| **M58** | feces | 24.300 −24.767 | 545.3394 [M + Na]^+^ 25.195 | 545.3449 | −10.09 | − | 567.3497 [M + HCOOH − H]^−^ | 567.3539 | −7.23 | − | 6 | C_30_H_50_O_7_ | 522.3557 | dehydrogenated trihydroxylated mogrol |

**Table S1.** *Cont*.

| **No.** | **Sample** | **t_R_ (min)** | **Measured Mass (Da) of [M + X]^+^** | **Predicated Mass (Da) of [M + X]^+^** | **Error (ppm)** | **Characteristic Positive Fragment Ions (Relative Abundance)** | **Measured Mass (Da) of [M + X]^−^** | **Predicated Mass (Da) of [M + X]^−^** | **Error (ppm)** | **Characteristic Negative Fragment Ions (Relative Abundance)** | **DBE** | **Molecular Formula** | **Exact Mass** | **Identification** |
| --- | --- | --- | --- | --- | --- | --- | --- | --- | --- | --- | --- | --- | --- | --- |
| **M59** | feces | 24.767 −25.683 | 545.3408 [M + Na]^+^ 25.565 | 545.3449 | −7.52 | − | 567.3508 [M + HCOOH − H]^−^ | 567.3539 | −5.46 | MS^2^: 521.3442(100), C_30_H_49_O_7_; 503.3303(6.26), C_30_H_47_O_6_; 463.3095(38.56), C_27_H_43_O_6_; 403.2823(60.52), C_25_H_39_O_4_. | 6 | C_30_H_50_O_7_ | 522.3557 | dehydrogenated trihydroxylated mogrol |
| **M60** | feces | 26.933 −27.233 | − | − | − | − | 567.3499 [M + HCOOH − H]^−^ | 567.3539 | −7.05 | MS^2^: 521.3433(26.35), C_30_H_49_O_7_; 491.3363(100), C_29_H_47_O_6_; 473.3127(16.90), C_29_H_45_O_5_; 455.3168(67.24), C_29_H_43_O_4_; 437.2922(4.43), C_29_H_41_O_3_; 411.2930(4.43), C_27_H_39_O_3_;  MS^3^(491.3364: 455.3151(100), C_29_H_43_O_4_; 437.29221(100), C_29_H_41_O_3_; 411.2930(59.52), C_27_H_39_O_3_. | 6 | C_30_H_50_O_7_ | 522.3557 | dehydrogenated trihydroxylated mogrol |
| **M61** | feces | 27.550 −28.083 | − | − | − | − | 567.3498 [M + HCOOH − H]^−^ | 567.3539 | −7.23 | − | 6 | C_30_H_50_O_7_ | 522.3557 | dehydrogenated trihydroxylated mogrol |

**Table S1.** *Cont*.

| **No.** | **Sample** | **t_R_ (min)** | **Measured Mass (Da) of [M + X]^+^** | **Predicated Mass (Da) of [M + X]^+^** | **Error (ppm)** | **Characteristic Positive Fragment Ions (Relative Abundance)** | **Measured Mass (Da) of [M + X]^−^** | **Predicated Mass (Da) of [M + X]^−^** | **Error (ppm)** | **Characteristic Negative Fragment Ions (Relative Abundance)** | **DBE** | **Molecular Formula** | **Exact Mass** | **Identification** |
| --- | --- | --- | --- | --- | --- | --- | --- | --- | --- | --- | --- | --- | --- | --- |
| **M62** | feces | 24.117 −24.733 |  |  |  |  | 565.3351  [M + HCOOH − H]^−^ 555.3059 [M + Cl − H]^−^ | 565.3382 555.3094 | −5.48 6.30 | MS^2^: 519.3290(22.53), C_30_H_47_O_7_; 501.3169(1.40), C_30_H_45_O_6_; 417.2612(100), C_25_H_37_O_5_. MS^3^(417.2612): 399.2536(61.28), C_25_H_35_O_4_; 381.2447(100), C_22_H_37_O_5_; 363.2262(90.23), C_22_H_36_O_4_. |  | C_30_H_48_O_7_ | 520.3400 | didehydrogenated trihydroxylated mogrol |
| **M63** | feces | 25.883 −26.417 |  |  |  |  | 565.3357 [[M + HCOOH − H]^−^ | 565.3382 | −4.42 | MS^2^: 519.3286(24.093), C_30_H_47_O_7_; 501.3212(11.40), C_30_H_45_O_6_; 417.2611(100), C_25_H_37_O_5_; 399.2537(84.72), C_25_H_35_O_4_; 381.2378(9.04), C_22_H_37_O_5_; 203.1183(5.56), C_10_H_19_O_4_. MS^3^(417.2612): 399.2447(57.22), C_25_H_35_O_4_; 381.2306(100), C_22_H_37_O_5_; 203.1206(57.22), C_10_H_19_O_4_. | 7 | C_30_H_48_O_7_ | 520.3044 | didehydrogenated trihydroxylated mogrol |

**Table S1.** *Cont*.

| **No.** | **Sample** | **t_R_ (min)** | **Measured Mass (Da) of [M + X]^+^** | **Predicated Mass (Da) of [M + X]^+^** | **Error (ppm)** | **Characteristic Positive Fragment Ions (Relative Abundance)** | **Measured Mass (Da) of [M + X]^−^** | **Predicated Mass (Da) of [M + X]^−^** | **Error (ppm)** | **Characteristic Negative Fragment Ions (Relative Abundance)** | **DBE** | **Molecular Formula** | **Exact Mass** | **Identification** |
| --- | --- | --- | --- | --- | --- | --- | --- | --- | --- | --- | --- | --- | --- | --- |
| **M64** | feces | 28.533 −28.900 | − | − | − | − | 565.3357 [M+HCOOH−H]^−^ | 565.3382 | −2.48 | MS^2^: 519.3238(2.79), C_30_H_47_O_7_; 461.2890(100), C_28_H_45_O_5_; 443.2755(28.28), C_27_H_39_O_5_; MS^3^(461.2889): 443.2717(100), C_27_H_39_O_5_. | 7 | C_30_H_48_O_7_ | 520.3044 | didehydrogenated trihydroxylated mogrol |
| **M65** | feces | 30.150 −30.700 | − | − | − | − | 565.3347 [M + HCOOH − H]^−^ | 565.3382 | −6.19 | MS^2^: 519.3242(5.59), C_30_H_47_O_7_; 461.2869(43.40), C_28_H_45_O_5_; 443.2826(55.09), C_27_H_39_O_5_. MS^3^(425.2700): 189.0910(100), C_9_H_17_O_4_. | 7 | C_30_H_48_O_7_ | 520.3044 | didehydrogenated trihydroxylated mogrol |
| **M66** | feces | 13.633 −14.083 | − | − | − | − | 585.3613 [M + HCOOH − H]^−^ | 585.3644 | −5.30 | MS^2^: 539.3583(74.00), C_30_H_51_O_8_; 521.3452(100), C_30_H_49_O_7_; 503.3313(87.00), C_30_H_47_O_6_; 485.3180(74.00), C_30_H_45_O_5_; 379.2626(87.00), C_26_H_35_O_2_. | 5 | C_30_H_52_O_8_ | 540.3662 | tetrahydroxylated mogrol |
| **M67** | feces | 14.083 −14.433 | 563.3491 [M + Na]^+^ 13.952 | 563.3554 | −11.18 | − | 585.3601 [M + HCOOH − H]^−^ | 585.3644 | −7.35 | − | 5 | C_30_H_52_O_8_ | 540.3662 | tetrahydroxylated mogrol |
| **M68** | feces | 14.433 −14.900 | − | − | − | − | 585.3608 [M + HCOOH − H]^−^ | 585.3644 | −6.15 | − | 5 | C_30_H_52_O_8_ | 540.3662 | tetrahydroxylated mogrol |

**Table S1.** *Cont*.

| **No.** | **Sample** | **t_R_ (min)** | **Measured Mass (Da) of [M + X]^+^** | **Predicated Mass (Da) of [M + X]^+^** | **Error (ppm)** | **Characteristic Positive Fragment Ions (Relative Abundance)** | **Measured Mass (Da) of [M + X]^−^** | **Predicated Mass (Da) of [M + X]^−^** | **Error (ppm)** | **Characteristic Negative Fragment Ions (Relative Abundance)** | **DBE** | **Molecular Formula** | **Exact Mass** | **Identification** |
| --- | --- | --- | --- | --- | --- | --- | --- | --- | --- | --- | --- | --- | --- | --- |
| **M69** | feces | 18.083 −18.617 | 563.3564 [M + Na]^+^ | 563.3554 | 1.78 | − | 585.3603 [M + HCOOH − H]^−^ | 585.3644 | −4.95 | − | 5 | C_30_H_52_O_8_ | 540.3662 | tetrahydroxylated mogrol |
| **M70** | feces | 19.050 −19.667 | − | − | − | − | 585.3613 [M+HCOOH−H]^−^ | 585.3644 | −5.30 | − | 5 | C_30_H_52_O_8_ | 540.3662 | tetrahydroxylated mogrol |
| **M71** | feces | 15.317 −15.800 | − | − | − | − | 583.3444 [M + HCOOH − H]^−^ | 583.3488 | −7.54 | − | 6 | C_30_H_50_O_8_ | 538.3506 | dehydrogenated tetrahydroxylated mogrol |
| **M72** | feces | 15.800 −16.133 | 561.3375 [M + Na]^+^ 18.352 | 561.3398 | −4.10 | − | 583.3454 [M + HCOOH − H]^−^ | 583.3488 | −8.54 | − | 6 | C_30_H_50_O_8_ | 538.3506 | mogrol+4O−2H |
| **M73** | feces | 20.133 −20.633 | − | − | − | − | 583.3454 [M + HCOOH − H]^−^ | 583.3488 | −6.00 | MS^2^: 537.3423(100), C_30_H_49_O_8_; 519.3309(7.99), C_30_H_47_O_7_; 501.3288(19.20), C_30_H_45_O_6_; 417.2687(6.512), C_25_H_37_O_5_; 413.2735(0.77), C_26_H_37_O_4_; 431.2760(9.72), C_26_H_39_O_5_. MS^3^(538.3425): 485.27.63(7.08), C_29_H_41_O_6_; 431.27728(100), C_26_H_39_O_5_; 413.2658(7.08), C_26_H_37_O_4_; 395.2521(20.05), C_26_H_35_O_3_. | 6 | C_30_H_50_O_8_ | 538.3506 | dehydrogenated tetrahydroxylated mogrol |

**Table S1.** *Cont*.

| **No.** | **Sample** | **t_R_ (min)** | **Measured Mass (Da) of [M + X]^+^** | **Predicated Mass (Da) of [M + X]^+^** | **Error (ppm)** | **Characteristic Positive Fragment Ions (Relative Abundance)** | **Measured Mass (Da) of [M + X]^−^** | **Predicated Mass (Da) of [M + X]^−^** | **Error (ppm)** | **Characteristic Negative Fragment Ions (Relative Abundance)** | **DBE** | **Molecular Formula** | **Exact Mass** | **Identification** |
| --- | --- | --- | --- | --- | --- | --- | --- | --- | --- | --- | --- | --- | --- | --- |
| **M74** | feces | 20.608 −21.075 | − | − | − | − | 583.3465 [M + HCOOH − H]^−^ | 583.3488 | −3.94 | MS^2^: 537.34086(53.58), C_30_H_49_O_8_; 519.3298(7.01), C_30_H_47_O_7_; 479.2977(68.14), C_27_H_43_O_7_; 471.3132(4.07), C_29_H_43_O_5_; 419.2762(100), C_25_H_39_O_5_; 417.2602(66.51), C_25_H_37_O_5_; 399.2551(3.69), C_25_H_35_O_4_; 383.2556(10.25), C_25_H_35_O_3_. MS^3^(479.2975): 383.2587(100), C_24_H_31_O_4_; 419.2824(62.35), C_25_H_39_O_5_; 417.2572(54.71), C_25_H_37_O_5_. | 6 | C_30_H_50_O_8_ | 538.3506 | dehydrogenated tetrahydroxylated mogrol |
| **M75** | feces | 21.050 −21.717 | 561.3348 [M + Na]^+^ 20.625 | 561.3398 | −8.91 | − | 583.3465 [M + HCOOH − H]^−^ | 583.3488 | −3.94 | MS^2^: 537.3410(100), C_30_H_49_O_8_; 519.3374(7.567), C_30_H_47_O_7_; 501.30281(1.71), C_30_H_45_O_6_; 463.2929(1.71), C_27_H_43_O_6_; 443.2334(1.71), C_27_H_39_O_5_. | 6 | C_30_H_50_O_8_ | 538.3506 | dehydrogenated tetrahydroxylated mogrol |

**Table S1.** *Cont*.

| **No.** | **Sample** | **t_R_ (min)** | **Measured Mass (Da) of [M + X]^+^** | **Predicated Mass (Da) of [M + X]^+^** | **Error (ppm)** | **Characteristic Positive Fragment Ions (Relative Abundance)** | **Measured Mass (Da) of [M + X]^−^** | **Predicated Mass (Da) of [M + X]^−^** | **Error (ppm)** | **Characteristic Negative Fragment Ions (Relative Abundance)** | **DBE** | **Molecular Formula** | **Exact Mass** | **Identification** |
| --- | --- | --- | --- | --- | --- | --- | --- | --- | --- | --- | --- | --- | --- | --- |
| **M76** | feces | 22.483 −22.900 | 561.3369 [M + Na]^+^ 20.927 | 561.3398 | −5.17 | − | 583.3452 [M + HCOOH − H]^−^ | 583.3488 | −6.17 | − | 6 | C_30_H_50_O_8_ | 538.3506 | dehydrogenated tetrahydroxylated mogrol |
| **M77** | feces | 24.500 −24.967 | − | − | − | − | 583.3447 [M + HCOOH − H]^−^ | 583.3488 | −7.03 | − | 6 | C_30_H_50_O_8_ | 538.3506 | dehydrogenated tetrahydroxylated mogrol |
| **M78** | feces | 20.133 −21.100 | − | − | − | − | 581.3297 [M + HCOOH − H]^−^ | 581.3331 | −5.85 | − | 7 | C_30_H_48_O_8_ | 536.3349 | didehydrogenated tetrahydroxylated mogrol |
| **M79** | feces | 21.383 −22.233 | − | − | − | − | 581.3290 [M + HCOOH − H]^−^ | 581.3331 | −7.05 | MS^2^: 535.3233(3233), C_30_H_48_O_8;_ 477.2849(47.04), C_29_H_49_O_5_; 417.2588(100), C_25_H_37_O_5_; MS^3^(417.2588): 399.2555(58.17), C_25_H_35_O_4_; 381.2383(100), C_22_H_37_O_5_; 363.2252(74.84), C_22_H_36_O_4_. | 7 | C_30_H_48_O_8_ | 536.3349 | didehydrogenated tetrahydroxylated mogrol |
| **M80** | feces | 22.900 −23.150 | − | − | − | − | 581.3292 [M + HCOOH − H]^−^ | 581.3331 | −6.71 | − | 7 | C_30_H_48_O_8_ | 536.3349 | didehydrogenated tetrahydroxylated mogrol |

**Table S1.** *Cont*.

| **No.** | **Sample** | **t_R_ (min)** | **Measured Mass (Da) of [M + X]^+^** | **Predicated Mass (Da) of [M + X]^+^** | **Error (ppm)** | **Characteristic Positive Fragment Ions (Relative Abundance)** | **Measured Mass (Da) of [M + X]^−^** | **Predicated Mass (Da) of [M + X]^−^** | **Error (ppm)** | **Characteristic Negative Fragment Ions (Relative Abundance)** | **DBE** | **Molecular Formula** | **Exact Mass** | **Identification** |
| --- | --- | --- | --- | --- | --- | --- | --- | --- | --- | --- | --- | --- | --- | --- |
| **M81** | feces | 23.150 −23.800 | − | − | − | − | 581.3306 [M + HCOOH − H]^−^ 571.3024 [M + Cl − H]^−^ | 581.3331 571.3043 | −4.03 −3.33 | MS2: 535.3263(9.36), C_30_H_48_O_8_; 417.2615(100); 399.2525(45.12), C_25_H_35_O_4_; 383.2201(1.70), C_25_H_35_O_3_; 381.2419(4.05), C_22_H_37_O_5_. MS^3^(417.2616): 399.2535(100), C_25_H_35_O_4_; 381.2422(41.26), C_22_H_37_O_5_; 363.23692(4.07), C_22_H_36_O_4_; 203.1085(41.26), C_10_H_19_O_4_. | 7 | C_30_H_48_O_8_ | 536.3349 | didehydrogenated tetrahydroxylated mogrol |
| **M82** | feces | 23.800 −24.300 | − | − | − | − | 581.3308 [M + HCOOH − H]^−^ | 581.3331 | −3.96 | MS^2^: 535.3162(21.15), C_30_H_48_O_8_; 487.3035(20.09), C_30_H_47_O_5_; 417.2644(100), C_25_H_37_O_5_; 399.2525(43.21), C_25_H_35_O_4_. MS^3^(417.2644): 399.2515(100), C_25_H_35_O_4_; 381.2398(54.74), C_22_H_37_O_5_. | 7 | C_30_H_48_O_8_ | 536.3349 | didehydrogenated tetrahydroxylated mogrol |
| **M83** | feces | 25.433 −25.867 | − | − | − | − | 581.3304 [M + HCOOH − H]^−^ | 581.3331 | −4.64 | − | 7 | C_30_H_48_O_8_ | 536.3349 | didehydrogenated tetrahydroxylated mogrol |
| **M84** | feces | 25.867 −26.350 | − | − | − | − | 581.3291 [M + HCOOH − H]^−^ | 581.3331 | −4.00 | − | 7 | C_30_H_48_O_8_ | 536.3349 | didehydrogenated tetrahydroxylated mogrol |

**Table S1.** *Cont*.

| **No.** | **Sample** | **t_R_ (min)** | **Measured Mass (Da) of [M + X]^+^** | **Predicated Mass (Da) of [M + X]^+^** | **Error (ppm)** | **Characteristic Positive Fragment Ions (Relative Abundance)** | **Measured Mass (Da) of [M + X]^−^** | **Predicated Mass (Da) of [M + X]^−^** | **Error (ppm)** | **Characteristic Negative Fragment Ions (Relative Abundance)** | **DBE** | **Molecular Formula** | **Exact Mass** | **Identification** |
| --- | --- | --- | --- | --- | --- | --- | --- | --- | --- | --- | --- | --- | --- | --- |
| **M85** | feces | 17.467 −18.083 | − | − | − | − | 587.2977 [M + Cl − H]^−^ 597.3263 [M + HCOOH − H]^−^ | 587.2992 597.3280 | −2.55 −2.85 | MS^2^: 551.3126(4.43), C_30_H_48_O_9_; 433.2617(3.80), C_27_H_45_O_4;_ 417.2600(100), C_25_H_37_O_5_; 399.2515(80.75), C_25_H_35_O_4_; 383.2116(3.80), C_25_H_35_O_3_; 381.2367(9.42), C_22_H_37_O_5_. MS^3^(417.2600): 399.2508(100), C_25_H_35_O_4_; 381.2398(29.63), C_22_H_37_O_5_; 203.1018(21.10), C_10_H_19_O_4_. | 7 | C_30_H_48_O_9_ | 552.3298 | didehydrogenated pentahydroxylated mogrol |
| **M86** | feces | 18.250 −19.000 | − | − | − | − | 587.2983 [M + Cl − H]^−^ 597.3257 [M + HCOOH − H] | 587.2992 597.3280 | 1.53 −3.85 | MS^2^: 415.2516(100), C_27_H_43_O_3_; 403.2472(18.34), C_25_H_39_O_4_; 397.2357(75.13), C_26_H_37_O_3_; 203.0987(13.76), C_10_H_19_O_4_. MS^3^(415.2516): 379.2178(100), C_26_H_35_O_2_. | 7 | C_30_H_48_O_9_ | 552.3298 | didehydrogenated pentahydroxylated mogrol |

^a^ Confirmed by comparison with reference compounds. DBE, Double bond equivalent.

**M4**

**M3**

**M0**

**M5**

|  | **t_R_ (min)** | **Peak Start** | **Peak End** | ***m*/*z*** | **Area** |
| --- | --- | --- | --- | --- | --- |
| **M0** | 24.966 | 24.770 | 25.078 | 1169.5900 | 9,489,561 |
| **M3** | 25.345 | 25.285 | 25.650 | 1169.5900 | 760,580 |
| **M4** | 25.960 | 25.650 | 25.933 | 1169.5900 | 545,593 |
| **M5** | 26.696 | 26.517 | 26.950 | 1169.5900 | 1,984,320 |

**Figure S1.** EICs at *m*/*z* 1169.59 of drug-containing feces (**above**) and blank feces (**below**).

**M1**

**M2**

**Figure S2.** *Cont*.

**M2**

**M1**

|  | **t_R_ (min)** | **Peak Start** | **Peak End** | ***m*/*z*** | **Area** |
| --- | --- | --- | --- | --- | --- |
| **M1** | 25.698 | 25.500 | 25.817 | 1285.6400 | 9,555,900 |
| **M2** | 26.043 | 25.817 | 26.300 | 1285.6400 | 15,997,951 |

**Figure S2.** EICs at *m*/*z* 1285.64 of drug-containing urine (**above**) and blank urine (**below**).

**M6**

|  | **t_R_ (min)** | **Peak Start** | **Peak End** | ***m*/*z*** | **Area** |
| --- | --- | --- | --- | --- | --- |
| **M6** | 25.923 | 25.753 | 26.103 | 1167.5800 | 24,868,935 |

**Figure S3.** EICs at *m*/*z* 1167.58 of drug-containing urine (**above**) and blank urine (**below**).

**M7**

|  | **t_R_ (min)** | **Peak Start** | **Peak End** | ***m*/*z*** | **Area** |
| --- | --- | --- | --- | --- | --- |
| **M7** | 25.983 | 25.800 | 26.117 | 1153.6000 | 1,610,485 |

**Figure S4.** EICs at *m*/*z* 1153.60 of drug-containing urine (**above**) and blank urine (**below**).

**M11**

**M12**

**M10**

**M9**

**M8**

**Figure S5.** *Cont*.

|  | **t_R_ (min)** | **Peak Start** | **Peak End** | ***m*/*z*** | **Area** |
| --- | --- | --- | --- | --- | --- |
| **M8** | 26.803 | 26.717 | 27.050 | 1007.5400 | 686,710 |
| **M9** | 27.173 | 27.050 | 27.633 | 1007.5400 | 5,499,380 |
| **M10** | 30.217 | 30.017 | 30.400 | 1007.5400 | 25,071,251 |
| **M11** | 30.525 | 30.400 | 30.767 | 1007.5400 | 6,584,321 |
| **M12** | 30.940 | 30.767 | 31.383 | 1007.5400 | 7,864,503 |

**Figure S5.** EICs at *m*/*z* 1007.54 of drug-containing feces (**above**) and blank feces (**below**).

**M13**

|  | **t_R_ (min)** | **Peak Start** | **Peak End** | ***m*/*z*** | **Area** |
| --- | --- | --- | --- | --- | --- |
| **M13** | 26.927 | 26.800 | 27.117 | 1005.53700 | 5,673,582 |

**Figure S6.** EICs at *m*/*z* 1005.53 of drug-containing feces (**above**) and blank feces (**below**).

**M14**

**Figure S7.** *Cont*.

|  | **t_R_ (min)** | **Peak Start** | **Peak End** | ***m*/*z*** | **Area** |
| --- | --- | --- | --- | --- | --- |
| **M14** | 27.728 | 27.383 | 28.000 | 991.5500 | 4,700,498 |

**Figure S7.** EICs at *m*/*z* 991.55 of drug-containing feces (**abov**e) and blank feces (**below**).

**M19**

**M18**

**M17**

**M16**

**M15**

|  | **t_R_ (min)** | **Peak Start** | **Peak End** | ***m*/*z*** | **Area** |
| --- | --- | --- | --- | --- | --- |
| **M15** | 29.365 | 29.183 | 29.717 | 845.4900 | 122,469,048 |
| **M16** | 30.648 | 30.467 | 30.883 | 845.4900 | 10,029,179 |
| **M17** | 31.775 | 31.433 | 32.333 | 845.4900 | 14,873,427 |
| **M18** | 33.298 | 33.000 | 33.500 | 845.4900 | 131,377,697 |
| **M19** | 33.905 | 33.500 | 34.100 | 845.4900 | 45,423,120 |

**Figure S8.** EICs at *m*/*z* 845.49 of drug-containing feces (**above**) and blank feces (**below**).

**M21**

**M20**

|  | **t_R_ (min)** | **Peak Start** | **Peak End** | ***m*/*z*** | **Area** |
| --- | --- | --- | --- | --- | --- |
| **M20** | 29.908 | 29.847 | 30.278 | 843.4700 | 50,669,383 |
| **M21** | 33.604 | 33.350 | 33.917 | 843.4700 | 50,269,416 |

**Figure S9.** EICs at *m*/*z* 843.47 of drug-containing feces (**above**) and blank feces (**below**).

M22

**Figure S10.** *Cont*.

|  | **t_R_ (min)** | **Peak start** | **Peak end** | ***m*/*z*** | **Area** |
| --- | --- | --- | --- | --- | --- |
| **M22** | 34.028 | 33.700 | 34.517 | 829.5000 | 162025962 |

**Figure S10.** EICs at *m*/*z* 829.50 of drug-containing feces (**above**) and blank feces (**below**).

**M23**

|  | **t_R_ (min)** | **Peak Start** | **Peak End** | ***m*/*z*** | **Area** |
| --- | --- | --- | --- | --- | --- |
| **M23** | 34.813 | 34.567 | 35.183 | 827.4800 | 29,484,814 |

**Figure S11.** EICs at *m*/*z* 827.48 of drug-containing feces (**above**) and blank feces (**below**).

**M25**

**M24**

|  | **t_R_ (min)** | **Peak start** | **Peak end** | ***m*/*z*** | **Area** |
| --- | --- | --- | --- | --- | --- |
| **M24** | 34.997 | 34.767 | 35.433 | 683.4400 | 180,532,696 |
| **M25** | 37.507 | 36.950 | 37.983 | 683.4400 | 896,803,169 |

**Figure S12.** EICs at *m*/*z* 683.44 of drug-containing feces (**above**) and blank feces (**below**).

**A**

**M27**

**M26**

**B**

|  | **t_R_ (min)** | **Peak Start** | **PEAK End** | ***m*/*z*** | **Area** |
| --- | --- | --- | --- | --- | --- |
| **M26** | 36.120 | 35.883 | 36.500 | 681.4200 | 48,856,108 |
| **M27** | 38.860 | 38.433 | 39.400 | 681.4200 | 790,013,598 |

**Figure S13.** EICs at *m*/*z* 681.42 of drug-containing feces (**above**) and blank feces (**below**).

**A**

**M29**

**M28**

**C**

**B**

|  | **t_R_ (min)** | **Peak Start** | **Peak End** | ***m*/*z*** | **Area** |
| --- | --- | --- | --- | --- | --- |
| **M28** | 45.978 | 45.683 | 46.233 | 521.3800 | 92,475,813 |
| **M29** | 46.467 | 46.233 | 47.133 | 521.38 | 646,804,735 |

**Figure S14.** EICs at *m*/*z* 521.38 of drug-containing feces (**above**) and blank feces (**below**).

**A**

**M31**

**M30**

**B**

**Figure S15.** *Cont*.

|  | **t_R_ (min)** | **Peak start** | **Peak end** | ***m*/*z*** | **Area** |
| --- | --- | --- | --- | --- | --- |
| **M30** | 52.478 | 52.000 | 52.667 | 519.3700 | 24,183,728 |
| **M31** | 52.953 | 52.667 | 53.383 | 519.3700 | 324,786,331 |

**Figure S15.** EICs at *m*/*z* 519.37 of drug-containing feces (**above**) and blank feces (**below**).

**M34**

**M33**

**M35**

**M32**

**A**

**B**

|  | **t_R_ (min)** | **Peak Start** | **PEAK End** | ***m*/*z*** | **Area** |
| --- | --- | --- | --- | --- | --- |
| **M32** | 23.007 | 22.600 | 23.433 | 553.3700 | 390,217,566 |
| **M33** | 26.528 | 26.300 | 26.767 | 553.3700 | 46,223,582 |
| **M34** | 27.482 | 27.183 | 27.750 | 553.3700 | 125,631,723 |
| **M35** | 28.152 | 27.967 | 28.633 | 553.3700 | 488,223,880 |

**Figure S16.** EICs at t *m*/*z* 553.37 of drug-containing feces (**above**) and blank feces (**below**).

**M36**

**M38**

**M41**

**M40**

**M39**

**M37**

**Figure S17.** *Cont*.

|  | **t_R_ (min)** | **Peak Start** | **Peak End** | ***m*/*z*** | **Area** |
| --- | --- | --- | --- | --- | --- |
| **M36** | 26.475 | 26.117 | 26.767 | 551.3600 | 33,006,739 |
| **M37** | 27.050 | 26.767 | 27.367 | 551.3600 | 23,418,337 |
| **M38** | 29.487 | 29.183 | 29.683 | 551.3600 | 46,101,388 |
| **M39** | 30.887 | 30.283 | 31.250 | 551.3600 | 27,487,975 |
| **M40** | 31.537 | 31.250 | 31.833 | 551.3600 | 20,088,773 |
| **M41** | 33.122 | 32.867 | 33.367 | 551.3600 | 27,408,608 |

**Figure S17.** EICs at *m*/*z* 551.36 of drug-containing feces (**above**) and blank feces (**below**).

**M48**

**M49**

**M50**

**M47**

**M46**

**M45**

**M44**

**M42**

**M43**

**Figure S18.** *Cont*.

|  | **t_R_ (min)** | **Peak Start** | **Peak End** | ***m*/*z*** | **Area** |
| --- | --- | --- | --- | --- | --- |
| **M42** | 16.305 | 16.117 | 16.500 | 569.3700 | 12,435,301 |
| **M43** | 16.728 | 16.500 | 17.033 | 569.3700 | 77,259,589 |
| **M44** | 17.280 | 17.033 | 17.767 | 569.3700 | 284,733,513 |
| **M45** | 18.140 | 17.767 | 18.617 | 569.3700 | 44,372,830 |
| **M46** | 18.923 | 18.617 | 19.333 | 569.3700 | 89,188,731 |
| **M47** | 21.464 | 21.150 | 21.650 | 569.3700 | 108,520,771 |
| **M48** | 21.755 | 21.650 | 21.933 | 569.3700 | 30,986,855 |
| **M49** | 22.121 | 21.933 | 22.300 | 569.3700 | 34,753,952 |
| **M50** | 22.531 | 22.300 | 22.700 | 569.3700 | 53,158,418 |

**Figure S18.** EICs at *m*/*z* 569.37 of drug-containing feces (**above**) and blank feces (**below**).

**M52**

**M51**

|  | **t_R_ (min)** | **Peak Start** | **Peak End** | ***m*/*z*** | **Area** |
| --- | --- | --- | --- | --- | --- |
| **M51** | 11.410 | 11.233 | 11.783 | 567.3500 | 15908470 |
| **M52** | 18.482 | 18.233 | 18.833 | 567.3500 | 43804798 |

**Figure S19.** EICs at *m*/*z* 567.35 of drug-containing kidney (**above**) and blank kidney (**below**).

**M58**

**M59**

**M57**

**M60**

**M61**

**M54**

**M55**

**M56**

**M53**

|  | **t_R_ (min)** | **Peak Start** | **PEAK End** | ***m*/*z*** | **Area** |
| --- | --- | --- | --- | --- | --- |
| **M53** | 19.893 | 19.650 | 20.483 | 567.3500 | 463,948,611 |
| **M54** | 20.977 | 20.750 | 21.233 | 567.3500 | 21,448,937 |
| **M55** | 21.761 | 21.467 | 22.000 | 567.3500 | 79,454,258 |
| **M56** | 22.365 | 22.000 | 22.550 | 567.3500 | 79,252,367 |
| **M57** | 24.123 | 23.867 | 24.300 | 567.3500 | 39,700,636 |
| **M58** | 24.478 | 24.300 | 24.767 | 567.3500 | 86,848,220 |
| **M59** | 25.380 | 24.767 | 25.683 | 567.3500 | 17,421,558 |
| **M60** | 27.050 | 26.933 | 27.233 | 567.3500 | 21,097,294 |
| **M61** | 27.728 | 27.550 | 28.083 | 567.3500 | 88,863,196 |

**Figure S20.** EICs at *m*/*z* 567.35 of drug-containing feces (**above**) and blank feces (**below**).

**M64**

**M62**

**M63**

**M65**

**Figure S21.** *Cont*.

|  | **t_R_ (min)** | **Peak Start** | **Peak End** | ***m*/*z*** | **Area** |
| --- | --- | --- | --- | --- | --- |
| **M62** | 24.412 | 24.117 | 24.733 | 565.34700 | 38,437,245 |
| **M63** | 26.052 | 25.883 | 26.417 | 565.34700 | 102,677,796 |
| **M64** | 28.810 | 28.533 | 28.900 | 565.34700 | 35,880,113 |
| **M65** | 30.340 | 30.150 | 30.700 | 565.34700 | 23,867,787 |

**Figure S21.** EICs at *m*/*z* 565.34 of drug-containing feces (**above**) and blank feces (**below**).

**M67**

**M70**

**M69**

**M68**

**M66**

|  | **t_R_ (min)** | **Peak Start** | **Peak End** | ***m*/*z*** | **Area** |
| --- | --- | --- | --- | --- | --- |
| **M66** | 13.872 | 13.633 | 14.083 | 585.3600 | 13,548,270 |
| **M67** | 14.242 | 14.083 | 14.433 | 585.3600 | 5,583,766 |
| **M68** | 14.603 | 14.433 | 14.900 | 585.3600 | 5,640,311 |
| **M69** | 18.307 | 18.083 | 18.617 | 585.3600 | 9,156,984 |
| **M70** | 19.408 | 19.050 | 19.667 | 585.3600 | 10,312,539 |

**Figure S22.** EICs at *m*/*z* 585.36 of drug-containing feces (**above**) and blank feces (**below**).

**M77**

**M76**

**M75**

**M74**

**M71**

**M73**

**M72**

|  | **t_R_ (min)** | **Peak Start** | **Peak End** | ***m*/*z*** | **Area** |
| --- | --- | --- | --- | --- | --- |
| **M71** | 15.573 | 15.317 | 15.800 | 583.3500 | 13,648,742 |
| **M72** | 15.997 | 15.800 | 16.133 | 583.3500 | 4,644,757 |
| **M73** | 20.492 | 20.133 | 20.633 | 583.3500 | 54,507,901 |
| **M74** | 20.800 | 20.608 | 21.075 | 583.3500 | 77,998,658 |
| **M75** | 21.453 | 21.050 | 21.717 | 583.3500 | 39,341,209 |
| **M76** | 22.895 | 22.483 | 22.900 | 583.3500 | 12,303,039 |
| **M77** | 24.710 | 24.500 | 24.967 | 583.3500 | 7,437,147 |

**Figure S23.** EICs at *m*/*z* 583.35 of drug-containing feces (**above**) and blank feces (**below**).

**M84**

**M83**

**M82**

**M81**

**M809**

**M79**

**M78**

|  | **t_R_ (min)** | **Peak Start** | **Peak End** | ***m*/*z*** | **Area** |
| --- | --- | --- | --- | --- | --- |
| **M78** | 20.615 | 20.133 | 21.100 | 581.3300 | 30,479,610 |
| **M79** | 21.815 | 21.383 | 22.233 | 581.3300 | 45,861,423 |
| **M80** | 23.007 | 22.900 | 23.150 | 581.3300 | 18,882,764 |
| **M81** | 23.433 | 23.150 | 23.800 | 581.3300 | 285,519,682 |
| **M82** | 23.988 | 23.800 | 24.300 | 581.3300 | 19,376,253 |
| **M83** | 25.682 | 25.433 | 25.867 | 581.3300 | 36,030,998 |
| **M84** | 25.990 | 25.867 | 26.350 | 581.3300 | 14,114,954 |

**Figure S24.** EICs at *m*/*z* 581.33 of drug-containing feces (**above**) and blank feces (**below**).

**M86**

**M85**

|  | **t_R_ (min)** | **Peak Start** | **Peak End** | ***m*/*z*** | **Area** |
| --- | --- | --- | --- | --- | --- |
| **M85** | 17.707 | 17.467 | 18.083 | 587.3000 | 30,984,346 |
| **M86** | 18.607 | 18.250 | 19.000 | 587.3000 | 50,436,190 |

**Figure S25.** EICs at *m*/*z* 587.30 of drug-containing feces (**above**) and blank feces (**below**).
